# Pre-Treatment BOC Expression as an Indicator of Lymphovascular Invasion and *In Vitro* Chemotherapeutic Response in Upper Tract Urothelial Carcinoma

**DOI:** 10.32604/or.2026.070837

**Published:** 2026-03-23

**Authors:** Yin-Lun Chang, Hao-Lun Luo, Jei-Ming Peng, Chang-Chun Hsiao

**Affiliations:** 1 Department of Urology, Kaohsiung Chang Gung Memorial Hospital and Chang Gung University College of Medicine, Kaohsiung, Taiwan; 2 Graduate Institute of Clinical Medical Sciences, College of Medicine, Chang Gung University, Taoyuan, Taiwan; 3 Institute for Translational Research in Biomedicine, Kaohsiung Chang Gung Memorial Hospital, Kaohsiung, Taiwan

**Keywords:** BOC cell adhesion associated, oncogene regulated gene, upper tract urothelial carcinoma, lymphovascular invasion, biomarker, chemotherapy sensitivity

## Abstract

**Background:**

Upper tract urothelial carcinoma (UTUC) is an aggressive malignancy with high recurrence rates. Lymphovascular invasion (LVI) predicts a poor prognosis, yet its molecular drivers remain unclear. BOC cell adhesion-associated, oncogene-regulated (BOC, also known as Brother of CDO [Cell adhesion molecule-Related/Down-regulated by Oncogenes]), a hedgehog-related cell surface receptor, may serve as a biomarker for tumor progression and chemotherapy response. The study aimed to investigate the role of BOC in UTUC and its potential to predict LVI and chemotherapy response.

**Methods:**

Sequencing (RNA-seq) of 10 stage III UTUC, treatment-naïve, fresh tissue samples identified BOC as a candidate biomarker, which was subsequently validated in 2 independent cohorts (n = 74). Functional assays using urothelial carcinoma cell lines assessed the impact of BOC knockdown on cell migration, proliferation, and drug sensitivity. Methylation-specific PCR (MSP) was used to identify potential regulatory sites influencing BOC expression, and immunohistochemistry (IHC) analysis was conducted to compare BOC levels in high- and low-grade bladder cancer.

**Results:**

BOC expression was significantly higher in patients with lymphovascular invasion (LVI+, *p* < 0.01). Knockdown of BOC markedly inhibited cancer cell migration, without affecting proliferation. BOC knockdown enhanced the efficacy of cisplatin and gemcitabine in UTUC cells, although clinical tissue data did not provide direct evidence of its role as a predictor of drug response. Methylation analysis identified key regulatory sites that may control BOC expression, and IHC confirmed increased BOC levels in high-grade bladder cancer, linking it to tumor aggressiveness.

**Conclusion:**

BOC may serve as a potential biomarker for predicting LVI and chemotherapy response in UTUC. Its involvement in cancer cell migration and association with high-grade tumors suggests its clinical relevance for prognosis and treatment stratification. Further validation in larger, multi-center studies is warranted.

## 1 Introduction

Upper tract urothelial carcinoma (UTUC) is a relatively rare but aggressive malignancy that arises in the renal pelvis and ureter [1,2]. Notably, Taiwan has a significantly higher and steadily increasing incidence of UTUC compared to many neighboring countries [3,4], suggesting potential environmental, genetic, or lifestyle factors contributing to its development [5,6]. Despite ongoing research into the risk factors and treatments for UTUC, many aspects of its progression remain poorly understood. One particularly critical yet underexplored factor is lymphovascular invasion (LVI), a pathological feature associated with increased tumor aggressiveness and worse clinical outcomes [7–10]. Identifying molecular determinants that drive LVI is crucial for improving prognosis prediction and treatment approaches for patients with UTUC.

Radical nephroureterectomy (RNU) remains the gold standard treatment for UTUC; however, its high recurrence rate underscores the need for additional treatments [11–13]. Neoadjuvant chemotherapy (NAC) and adjuvant chemotherapy have been introduced to improve patient outcomes, but their effectiveness varies among individuals [14–17]. A major challenge in UTUC management is the lack of reliable molecular markers that can predict chemotherapy response and LVI status, which limits the ability to personalize treatments [18]. Given the heterogeneous nature of UTUC, identifying specific genetic or epigenetic alterations associated with LVI and chemotherapy sensitivity is critical to refining treatment strategies and improving patient prognoses.

Recent studies have highlighted the role of genetic and epigenetic alterations in UTUC progression and therapeutic response [19–22]. Although various genes and pathways have been implicated in tumor aggressiveness, specific molecular markers that can serve as reliable predictors for LVI and chemotherapy sensitivity remain unclear. In this context, BOC cell adhesion-associated, oncogene-regulated (BOC, also known as Brother of CDO [Cell adhesion molecule-Related/Down-regulated by Oncogenes]), which encodes a cell surface receptor involved in hedgehog signaling and cellular adhesion [23–26], has emerged as a promising candidate. While its roles in the developmental processes of other malignancies have been documented, the significance of BOC expression in UTUC, particularly in treatment-naïve fresh tissue specimens, has not been fully elucidated.

Thus, the purpose of this study was to determine the role of BOC in UTUC and if its expression can predict LVI and chemotherapy response. The expression levels, methylation status, and functional impact of BOC on urothelial carcinoma cancer cell behavior were studied. By using treatment-naïve tissue specimens, our investigation minimized confounding effects induced by prior therapy, thereby providing a clearer insight into the intrinsic role of BOC in UTUC progression and its potential utility for guiding personalized treatment.

## 2 Materials and Methods

### 2.1 Tissue Samples

Ten upper tract urothelial carcinoma (UTUC) tissue samples were obtained from patients at Kaohsiung Chang Gung Memorial Hospital (KCGMH), Taiwan. All samples were collected from stage III UTUC patients as part of a previous study and were used for RNA sequencing analysis. Among the 10 samples, six were confirmed as lymphovascular invasion–positive (LVI+) and four as lymphovascular invasion–negative (LVI-). To further validate the association between BOC expression and lymphovascular invasion, an independent cohort of 30 UTUC tissue samples was collected from stage III UTUC patients at KCGMH. This validation set consisted of 15 LVI(+) and 15 LVI(-) samples and was used for subsequent expression analysis. In addition, a second validation cohort comprising 44 stage III UTUC tissue samples was collected from patients at KCGMH to further confirm the robustness of the findings. This cohort included 22 LVI(+) samples and 22 LVI(-) samples. All procedures were conducted under approval from the Chang Gung Medical Foundation Institutional Review Board (IRB numbers: 202300477B0). All tumor tissue specimens were treatment-naïve fresh samples obtained from patients prior to any therapeutic interventions.

### 2.2 Whole Transcriptome RNA Sequencing (RNA-Seq) and Quantitative Reverse Transcription PCR (qRT-PCR)

Gene expression analysis initially focused on RNA extracted from tumor tissues of 10 patients with stage III UTUC treated at KCGMH. These 10 samples were subjected to whole transcriptome sequencing to determine gene expression profiles. Subsequent samples were analyzed for gene expression using quantitative reverse transcription PCR (qRT-PCR).

#### 2.2.1 Whole-Transcriptome RNA-Seq Workflow

Total RNA was extracted from patient tissue samples using TRIzol reagent (Thermo Fisher Scientific, Waltham, MA, USA). Tissues were homogenized in 1 mL TRIzol, followed by incubation at room temperature for 5 min. After the addition of 200 μL chloroform, samples were vigorously shaken for 15 s and centrifuged at 12,000× *g* for 15 min at 4°C. The upper aqueous layer was transferred to a new tube, mixed with an equal volume of isopropanol, and centrifuged to pellet RNA. The pellet was washed with 75% ethanol, air-dried, and resuspended in RNase-free water. RNA purity (A260/A280) and concentration (ng/µL) were determined using a SimpliNano Biochrom spectrophotometer (Biochrom, Holliston, MA, USA). RNA integrity was verified with the Qsep 100 DNA/RNA Analyzer (BiOptic Inc., Taiwan). Only samples with acceptable purity and integrity were used for library preparation. One microgram of total RNA per sample was processed with the KAPA mRNA HyperPrep Kit (cat. no. KK8580; KAPA Biosystems, Roche, Basel, Switzerland) following the manufacturer’s instructions. mRNA was purified with magnetic oligo-dT beads and fragmented at high temperature in the presence of magnesium. First-strand cDNA synthesis was performed with random hexamer priming, followed by second-strand synthesis and A-tailing to ensure strand specificity. Adapter ligation and size selection (300–400 bp) were conducted using the KAPA Pure Beads system. Libraries were amplified with KAPA HiFi HotStart ReadyMix, purified, and quality-checked using the Qubit 2.0 Fluorometer (Thermo Fisher Scientific, Waltham, MA, USA) and the Agilent 2100 Bioanalyzer. Sequencing was performed on an Illumina NovaSeq X platform (Illumina, San Diego, CA, USA) to generate 150 bp paired-end reads.

#### 2.2.2 Bioinformatic Processing and Differential Expression Analysis

Raw reads were converted to FASTQ files using CASAVA v1.8.2 and subjected to quality control with FastQC v0.11.9 and MultiQC v1.12. Adapter trimming and low-quality base removal were carried out with Trimmomatic v0.38. Clean reads were aligned to the human reference genome (GRCh38) using HISAT2 v2.2.0, and gene-level counts were obtained with featureCounts (Subread package, approximately v1.6). Normalization was performed according to experimental design: trimmed mean of M values (TMM) with ‘edgeR v3.36’ for datasets without biological replicates, and relative log expression (RLE) with ‘DESeq2 v1.34’ for datasets with replicates. Differentially expressed genes (DEGs) were identified using ‘DEGseq v1.48’ (for non-replicated data) or DESeq2 (for replicated data). Adjusted *p*-values were calculated using the Benjamini–Hochberg method to control the false discovery rate (FDR).

#### 2.2.3 Quantitative Reverse Transcription PCR (qRT-PCR) Validation

In the subsequent validation phase, 2 independent collections of clinical UTUC tissue samples were analyzed, consisting of 30 and 44 specimens, respectively. RNA was purified from these tissues using the QIAGEN RNA purification kit (cat. no. 74104; QIAGEN, Hilden, Germany). A total of 5 μg of RNA per sample was reverse-transcribed into complementary DNA (cDNA) using the PrimeScript^™^ RT Reagent Kit (cat. no. RR037A; TaKaRa, Shiga, Japan). The cDNA was then analyzed using qRT-PCR with SYBR Green PCR Master Mix on an ABI 7500 sequence detection system (Life Technologies, Carlsbad, CA, USA). Each reaction was performed in a final volume of 20 μL containing 50 ng of cDNA template, 0.25 μM of each forward and reverse primer, and 10 μL of 2× SYBR Green Master Mix. The thermal cycling program consisted of an initial denaturation at 95°C for 2 min, followed by 40 cycles of denaturation at 95°C for 30 s and annealing/extension at 55°C for 30 s. A melt curve analysis was subsequently performed from 60°C to 95°C with a 0.3°C increment per step to verify amplification specificity. All reactions were run in duplicate, and relative gene expression levels were calculated using the 2^−ΔΔCt^ method, with Actin serving as the internal control. Primers for qRT-PCR of BOC were: forward 5^′^-CGCACCTCCAAGACAGACTCAT-3^′^ and reverse 5^′^-TGGCTGGAATGCCAGAGATGGT-3^′^. Actin: forward 5^′^-CATGTACGTTGCTATCCAGGC-3^′^, reverse 5^′^-CTCCTTAATGTCACGCACGAT-3^′^.

### 2.3 Calculation of Relative Gene Expression and Public Database Analysis

To compare gene expression between patients positive for lymphovascular invasion (LVI+) and those negative for lymphovascular invasion (LVI-), relative expressions were calculated following a series of steps. First, the ΔCt value of each sample was determined by subtracting the Ct value of the internal control gene (Actin) from the Ct value of the target gene. This normalization process corrects for potential inter-sample variability, enhancing the comparability of expression results. Each ΔCt value was then converted into a relative expression value using the formula 2^−ΔCt^, a common approach in relative quantitative PCR that allows for linear scaling of gene expression levels. Next, the mean relative expression values for the LVI(−) and LVI(+) groups were calculated, providing an overall expression level for each group. Finally, the fold-change in gene expression was calculated by dividing the mean relative expression of the LVI(+) group by that of the LVI(−) group.

Patient survival and clinicopathological correlations were analyzed using publicly available datasets from The Cancer Genome Atlas Bladder Urothelial Carcinoma cohort (TCGA-BLCA). Overall survival–associated genes were identified using the Gene Expression Profiling Interactive Analysis (GEPIA) web tool (http://gepia2.cancer-pku.cn). Associations between gene expression and cancer stage or lymph node metastasis were evaluated using the University of Alabama at Birmingham Cancer Data Analysis Portal (UALCAN; http://ualcan.path.uab.edu), which also incorporates TCGA-BLCA transcriptomic data. Venn diagram analysis was performed to identify the overlapping genes among those significantly associated with tumor stage, nodal metastasis, overall survival, and oncogenic expression patterns. Transcript sequence information for BOC was obtained from the Ensembl database (https://www.ensembl.org), using the transcript ID ENST00000682979.1.

### 2.4 Cell Lines, shRNA, and qRT-PCR for Cancer Cell Proliferation, Migration, and Drug Sensitivity Assays

A series of experiments using UTUC and bladder cancer cell lines were performed to investigate if BOC is associated with cancer cell proliferation, metastasis, and chemotherapy sensitivity.

The BFTC909 (BCRC Number: 60069), BFTC905 (BCRC Number: 60068), and T24 (BCRC Number: 60062) cell lines were obtained from the American Type Culture Collection (ATCC, Manassas, VA, USA) and the Bioresource Collection and Research Center (BCRC, Hsinchu, Taiwan). All cell lines were authenticated by short tandem repeat (STR) profiling and were routinely tested and confirmed to be free of mycoplasma contamination. The BFTC909, BFTC905, and T24 cell lines were maintained as follows: BFTC909 cells in DMEM (Gibco, Thermo Fisher Scientific, Waltham, MA, USA) supplemented with 10% FBS; BFTC905 cells in RPMI-1640 (Gibco) with 15% FBS; and T24 cells in McCoy’s 5a Medium Modified (ATCC-formulated) with 10% FBS. All cultures were incubated at 37°C in a humidified 5% CO_2_ atmosphere. RNA knockdown was performed using shRNA virus from the RNAi Core at the Academia Sinica in Taiwan (https://rnai.genmed.sinica.edu.tw/en). The vectors were driven by a U6 promoter and carried a puromycin resistance cassette for antibiotic selection. The shRNA targeting sequences for BOC were shBOC#1: 5^′^-ACCTCCAAGACAGACTCATAT-3^′^, shBOC#2: 5^′^-CGACATTAAGATGCAGTGCTT-3^′^, and shBOC#3: 5^′^-ACACCACCTCTCACAATTTAG-3^′^. Two negative controls, ShScramble and ShVoid, which do not interact with any genes, were used as control groups.

Gene expression levels post-knockdown were measured using the same qRT-PCR method described earlier, employing SYBR Green PCR Master Mix and an ABI 7500 sequence detection system to quantify BOC expression and validate the efficacy of shRNA knockdown.

### 2.5 Boyden Chamber Cell Migration Assay

A total of 2 × 10^4^ BFTC909 cells were seeded in each well of the upper chamber (cat. no. TCS003024; JET Biofil, Guangzhou, China). For T24 cells, 3 × 10^4^ or 4 × 10^4^ cells per well were seeded under the same conditions. After 18 h, cell migration was assessed by staining with 0.1% crystal violet, and images were captured under an inverted fluorescence microscope (IX51; Olympus Corporation, Tokyo, Japan) at 100× magnification. Each experiment was conducted in triplicate. The stained cells were quantified using ImageJ software (version 1.8; NIH, Bethesda, MD, USA), and data were statistically analyzed using GraphPad Prism 10 (GraphPad, San Diego, CA, USA).

### 2.6 Real-Time Cell Migration and Proliferation Assay

The real-time cell migration and proliferation assay was conducted using the Real-Time Cell Analyzer (RTCA) system, following the method of Moniri et al. in 2015 [27]. In the migration assay, 20,000 BFTC909 cells were seeded in each well of a Cell Invasion and Migration (CIM)-plate (cat. no. 56-658-17001; ACEA Biosciences, Agilent Technologies, San Diego, CA, USA), and each experiment was conducted in triplicate. Cell migration was monitored using an xCELLigence RTCA instrument (Agilent, Santa Clara, CA, USA) over an 18-h period. Control experiments were also conducted, in which 160 µL of medium containing either Dulbecco’s Modified Eagle Medium (DMEM; Gibco, Thermo Fisher Scientific, Waltham, MA, USA) with 10% FBS was added to each well of the lower chamber, and 50 µL of serum-free medium was added to each well of the upper chamber. The plates were incubated at 37°C for 1 h, followed by the addition of 100 µL of medium containing 20,000 cancer cells to the upper chamber wells. After equilibrating at room temperature for 30 min, the test was started. After 18 h, the results were recorded using RTCA software (version 2.0; Agilent, Santa Clara, CA, USA).

For the cell proliferation assay, 5000 BFTC909 cells were seeded into each well of an E-Plate 16 (cat. no. 54-698-30001; Agilent Technologies, Santa Clara, CA, USA) and monitored over 96 h using the RTCA instrument. Each experiment was performed in triplicate. The protocol included 2 phases. First, a blank test was conducted every minute for a total of 11 measurements. In the second phase, cell measurements were taken every 15 min for a total of 400 measurements. For the blank test, each well of the E-plate was prepared by adding 50 μL of 12% DMEM. The experiment began by adding 150 μL of medium to each well containing the seeded 5000 cells. After the 96-h incubation, the results were quantified using impedance-based Cell Index values recorded by the RTCA system, and the data were analyzed statistically.

### 2.7 Drug Sensitivity Assay

BFTC909 cells were seeded in 96-well plates at 20,000 cells per well and incubated for 24 h. Each experiment was performed in triplicate. Paclitaxel (cat. no. T7402), gemcitabine (cat. no. G6423), cisplatin (cat. no. P4394), and epirubicin (cat. no. E9406) were purchased from Sigma-Aldrich (St. Louis, MO, USA) and added at concentrations ranging from 0.25 to 8 µM, with DMSO used as a control. To confirm the results, additional tests were conducted with certain drugs at lower concentrations. Both control and experimental group cells were maintained in puromycin (2 μg/mL; cat. no. A1113803; Gibco, Thermo Fisher Scientific, Waltham, MA, USA), which was discontinued 2–3 days before drug treatment. After 3 days of drug exposure, cell viability was assessed using the WST-1 Kit (cat. no. 11644807001; Roche Diagnostics GmbH, Mannheim, Germany). Results were statistically analyzed with GraphPad Prism 8 software (GraphPad, San Diego, CA, USA).

### 2.8 Identification of Methylation Sites in the BOC Gene

To identify methylation sites within the *BOC* gene sequence (ENST00000682979.1), the MethPrimer web tool (https://www.urogene.org/methprimer/) was used with the options “Pick MSP primers” and “Use CpG island prediction for primer selection.” The analysis parameters were set to Window = 100, Shift = 1, Obs/Exp = 0.6, and GC% = 50.

### 2.9 Methylation-Specific PCR (MSP)

For methylation analysis, a new set of samples from 13 patients was obtained. Chromosomal DNA was extracted from paired tumor and para-tumor tissues. Following DNA extraction, methylation analysis was performed to assess differences in methylation levels between tumor and para-tumor tissues.

Chromosomal DNA was extracted using the QIAamp DNA Mini Kit (cat. no. 51304; QIAGEN, Hilden, Germany). For DNA methylation analysis, 500 ng of DNA was treated using the EZ DNA Methylation Kit (cat. no. D5001; Zymo Research, Irvine, CA, USA). The reaction was performed for 16 h in the dark, during which time cytosine reacts with sodium bisulfite and is converted to uracil. The converted DNA was then subjected to a desulfonation reaction and eluted in 20 μL of elution buffer to yield methylated DNA. PCR amplification was conducted using the HotStarTaq^®^ MasterMix Kit (QIAGEN, Hilden, Germany, Cat. No. 203443) to detect CpG methylation, with primers designed by MethPrimer. PCR amplification conditions were: initial denaturation at 95°C for 1 min, followed by 40 cycles of 95°C for 30 s, 50°C for 30 s, and 72°C for 1 min, with a final extension at 72°C for 5 min.

Relative methylation levels at each CpG site were quantified using ImageJ software version 1.8 (NIH, Bethesda, MD, USA). The detection criterion required a clear band for the target gene in MSP analysis, with methylation differences observed between LVI(−) and LVI(+) samples, enabling discrimination between LVI(−) and LVI(+) based on methylation levels.

### 2.10 Immunohistochemistry

Immunohistochemistry (IHC) images of BOC in bladder urothelial carcinoma were obtained from The Cancer Genome Atlas (TCGA) bladder cohort through the Human Protein Atlas (https://www.proteinatlas.org/) to evaluate BOC expression across different tumor grades. The analysis included the staining of 4 low-grade and 7 high-grade tumor tissues.

Quantification of staining intensity and 3D surface plots was performed using ImageJ IHC Profiler version 1.53a (Wayne Rasband, National Institutes of Health, Bethesda, MD, USA). The original IHC images obtained from the Human Protein Atlas were imported into IHC Profiler using the “Cytoplasmic Stained Image” mode, and the H-DAB option was applied under “Color Deconvolution Vectors” to generate hematoxylin-enhanced visualizations. A uniform threshold value was applied across all samples to produce threshold segmentation maps. Regions of interest were outlined, the staining signal intensity was quantified, and 3D surface plots were generated to compare staining patterns between low-grade and high-grade tumors.

### 2.11 Statistical Analysis

Unless otherwise stated, all experiments were performed in at least three independent biological replicates, and data are presented as mean ± standard error of the mean (s.e.m.). For the Boyden chamber cell migration assay, statistical comparisons among multiple groups were conducted using one-way ANOVA followed by Tukey’s multiple comparisons test. For the real-time cell migration and proliferation assay (RTCA), statistical analysis was performed using repeated measures ANOVA followed by Tukey’s multiple comparisons test. For immunohistochemistry (IHC) analysis and qPCR-based comparisons of gene expression between LVI(+) and LVI(-) tumors, differences between the two groups were evaluated using two-tailed Student’s *t*-tests. Statistical significance was defined as *p* < 0.05. All statistical analyses were carried out using GraphPad Prism 10 (GraphPad, San Diego, CA, USA). Correlation analyses were performed using TCGA-BLCA RNA-seq data to investigate the association between BOC expression and metastasis-related genes. Spearman correlation coefficients were calculated, with statistical significance defined as *p* < 0.05.

## 3 Results

### 3.1 RNA-Seq Analysis and Candidate Gene Selection

**Figure 1:**
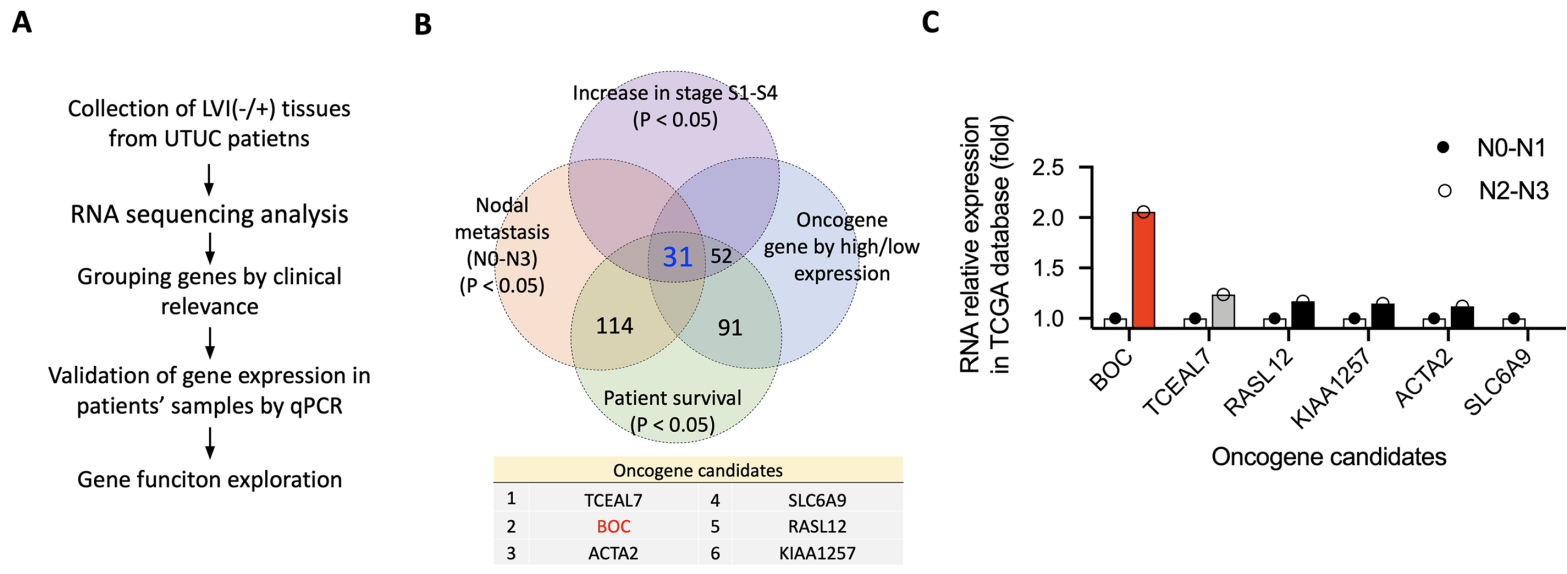
Identification and validation of oncogene candidates in UTUC. (**A**) Schematic workflow of the study, from LVI(–/+) tissue collection and RNA sequencing to gene selection, qRT-PCR validation, and functional analysis. (**B**) Venn diagram showing the overlap of genes significantly associated with tumor stage, nodal metastasis, survival, and oncogenic expression patterns. (**C**) RNA relative expression of six oncogene candidates in the TCGA BLCA dataset, comparing patients with N0–N1 vs. N2–N3 nodal metastasis.

RNA sequencing data from the 10 UTUC tissue samples were re-analyzed to identify differentially expressed genes associated with lymphovascular invasion (LVI). RNA was extracted from the tumor tissues and analyzed using RNA sequencing to identify any differences in gene expression between the LVI(+) and LVI(−) groups (Fig. 1A). Detailed information on the first analysis is reported in the prior study [28].

The significantly differentially expressed genes (DEGs) (*p* < 0.05) identified were further analyzed by cross-referencing with the TCGA-BLCA (Bladder Urothelial Carcinoma) database to identify genes associated with nodal metastasis, cancer stage, and patient survival in bladder urothelial carcinoma. Genes correlated with patient survival were identified using the Gene Expression Profiling Interactive Analysis (GEPIA) web tool [29], based on TCGA-BLCA data. Genes associated with cancer stage and nodal metastasis were analyzed using the University of Alabama at Birmingham Cancer Data Analysis Portal (UALCAN) web tool [30], which also uses TCGA-BLCA data. Genes with expression associated with any 2 stages (S1, S2, S3, and S4) or any 2 levels of metastasis (N0, N1, N2, and N3) were selected for analysis. Finally, 31 genes that overlapped were identified (Fig. 1B). Among these 31 genes, *BOC* exhibited the highest expression level in the N2-N3 nodal stage, exceeding its expression in N0-N1 (red bar in Fig. 1C). The expression level was also well above that of the second-ranked gene, *TCEAL7* (gray bar in Fig. 1C). The top 2-ranked genes, *BOC* and *TCEAL7*, were further validated in an independent set of UTUC patient samples.

### 3.2 Validation of the Association between BOC and TCEAL7 and Lymphovascular Invasion (LVI) Using Additional Tissue Samples from 30 Patients with Stage III UTUC

To validate the association of the genes *BOC* and *TCEAL7* and LVI, an independent collection of tissue samples from 30 patients hospitalized at KCGMH with Stage III UTUC was analyzed. There were 15 LVI(+) and 15 LVI(−) tissue samples, and the expression levels of *BOC* and *TCEAL7* were compared between these groups.

**Figure 2:**
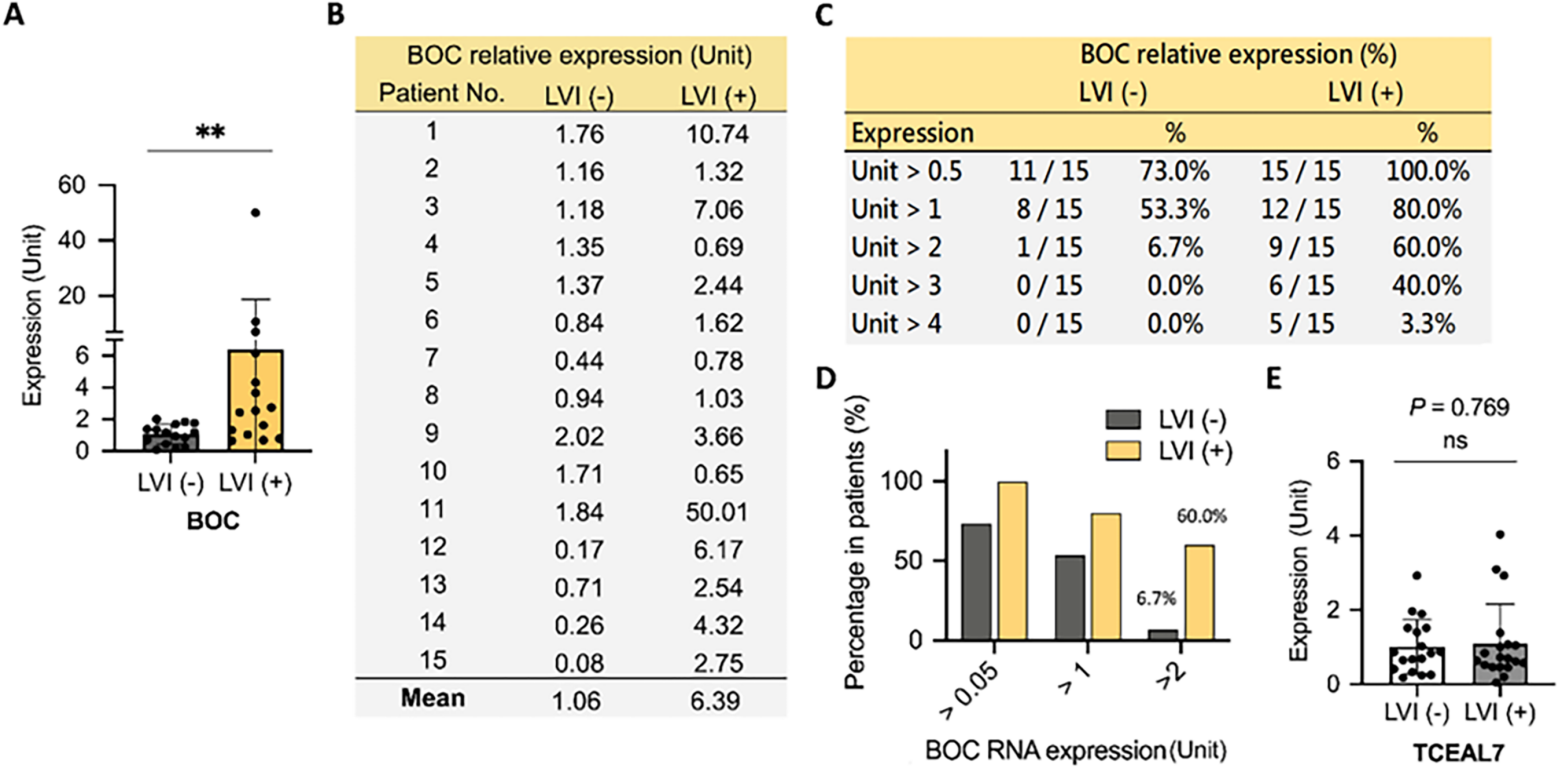
Elevated BOC gene expression is associated with LVI in UTUC. (**A**) Relative BOC expression was quantified by qRT-PCR in UTUC tumors with or without LVI. (**B**) Individual patient-level expression values of BOC in LVI(–) and LVI(+) tumors are shown. (**C**) Summary table indicating the percentage of patients with different fold-change thresholds of BOC expression in LVI(–) and LVI(+) groups. (**D**) Proportion of patients showing >0.5-, >1-, and >2-fold increases in BOC RNA expression between LVI(–) and LVI(+) groups. (**E**) Expression of TCEAL7 showed no significant difference between LVI(–) and LVI(+) tumors. All statistics were determined using a two-tailed Student’s *t*-test. **, *p* < 0.01; ns, not significant.

As shown in Fig. 2, the average expression level of *BOC* was significantly higher in LVI(+) patients compared to LVI(−) patients (approximately 6-fold higher, *p* = 0.0086) (Fig. 2A,B). Further analysis revealed that 53.3% of LVI(−) patients had relative *BOC* expression levels exceeding 1 unit, compared to 80.0% of LVI(+) patients (Fig. 2B,C). When the threshold was raised to 2 units, only 6.7% of LVI(−) patients exceeded this level, while 60.0% of LVI(+) patients surpassed this threshold (Fig. 2C). Using 2 units as the cutoff for *BOC* expression demonstrated its potential as an oncogenic biomarker specifically for LVI(+) UTUC (Fig. 2D). In contrast, analysis of *TCEAL7* showed no significant difference in expression levels between LVI(+) and LVI(−) UTUC (Fig. 2E).

These findings indicate the strong potential of *BOC* as a biomarker gene for distinguishing LVI status in patients with UTUC, whereas *TCEAL7* appears to lack discriminatory power in this context.

### 3.3 Second Validation of BOC as a Distinguishing Marker for Lymphovascular Invasion in 44 Patients with UTUC

**Figure 3:**
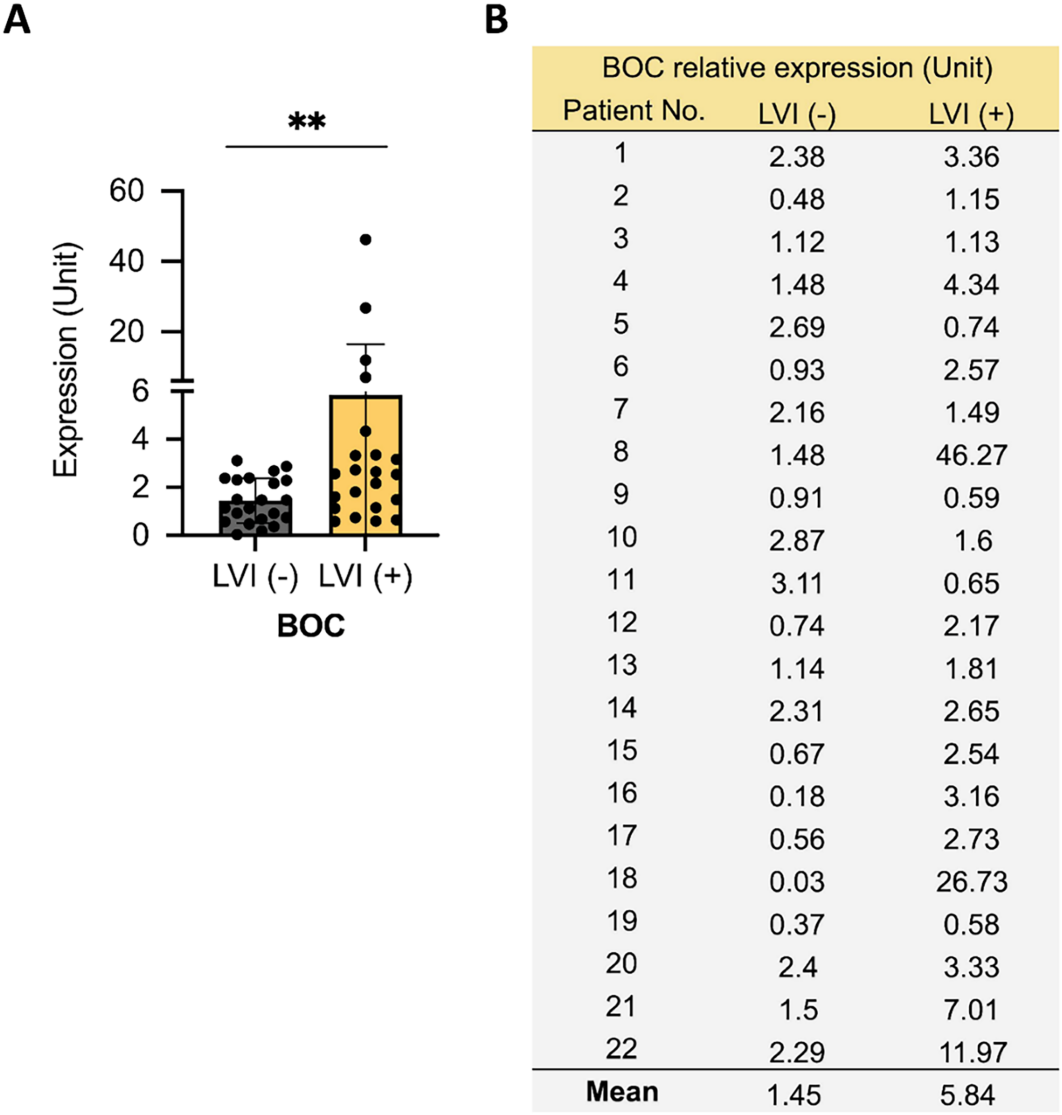
Association between BOC expression and LVI in a second UTUC patient cohort. (**A**) Relative BOC expression was quantified by qRT-PCR in UTUC tumors with or without LVI. (**B**) Individual patient-level BOC expression values are listed. All statistics were determined using a two-tailed Student’s *t*-test. **, *p* < 0.01.

To further confirm the ability of *BOC* to differentiate between LVI(+) and LVI(−) status in patients with UTUC, we conducted a second validation study with an additional set of 44 tissue samples collected from patients at KCGMH with Stage III UTUC. There were 22 LVI(+) samples and 22 LVI(−) samples. The results again demonstrated that the *BOC* gene expression level was significantly higher in the LVI(+) group compared to the LVI(−) group (an approximately 4-fold difference, *p* = 0.0093) (Fig. 3).

In these 2 validation phases, a total of 74 tissue samples were analyzed: 37 LVI(+) and 37 LVI(−). The finding consistently revealed that *BOC* exhibits excellent discriminatory power for differentiating patients with and without LVI (Figs. 2 and 3). These results highlight the strong potential of BOC as a biomarker to assist physicians in diagnosing LVI in patients with UTUC.

### 3.4 The Prognostic Value of BOC Expression in Cancer Patient Survival Analysis Using TCGA Data

**Figure 4:**
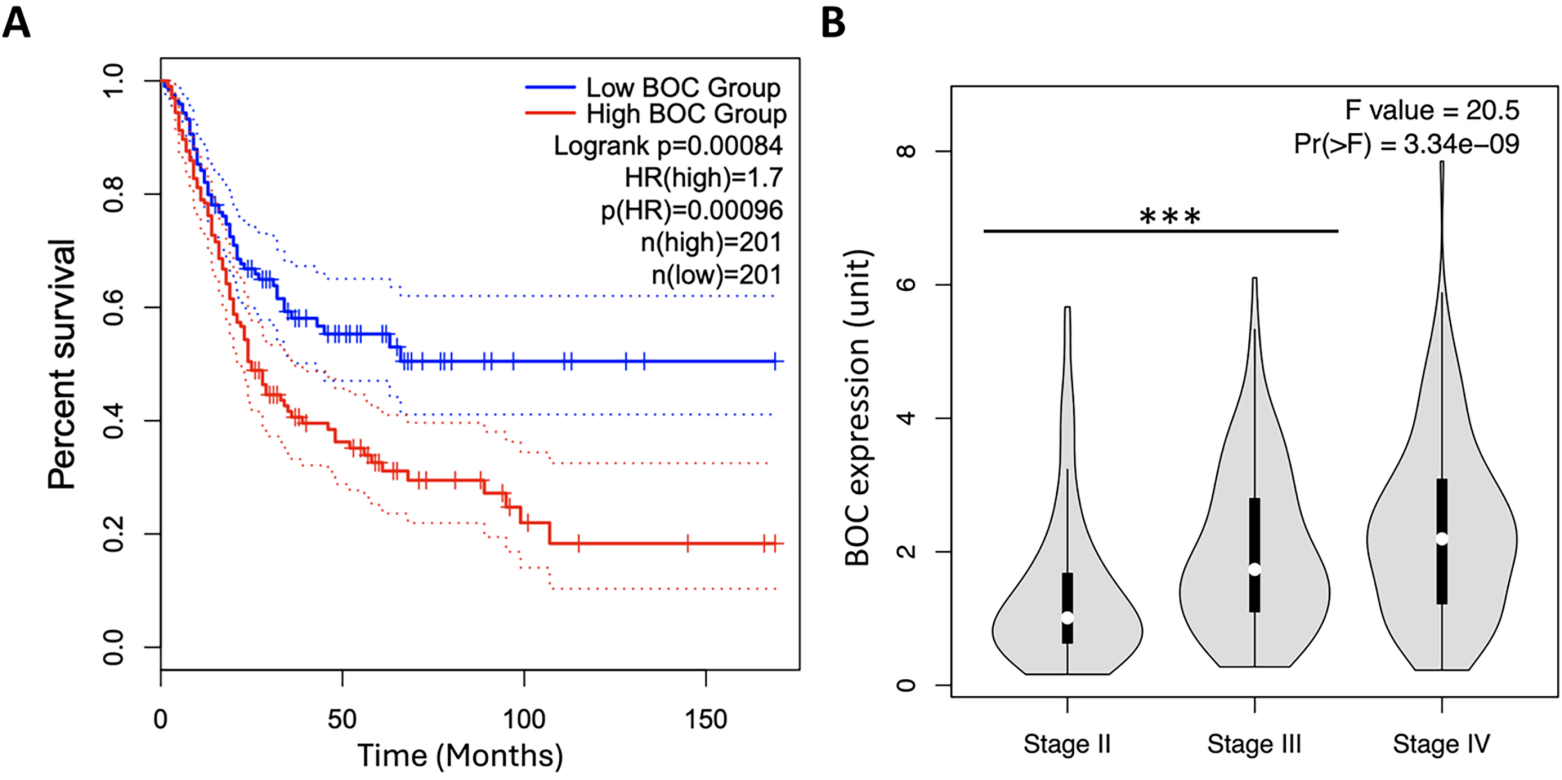
High BOC expression predicts poor survival and correlates with advanced tumor stage in the TCGA BLCA dataset. (**A**) Kaplan–Meier survival analysis of TCGA BLCA patients (n = 402) stratified by high vs. low BOC expression (log-rank *p* = 0.00084). (**B**) Violin plots of BOC expression across tumor stages (Stage II–IV, one-way ANOVA with Tukey’s post-hoc test, *p* = 3.34 × 10^−9^). ***, *p* < 0.001.

After confirming that *BOC* expression levels are elevated in LVI(+) UTUC patients, data from the TCGA database were analyzed using the GEPIA web tool (website: http://gepia.cancer-pku.cn) to investigate the impact of *BOC* gene expression on UTUC patient survival. The analysis revealed that patients with high *BOC* expression had significantly lower survival rates compared to those with low expression levels (*p* = 0.00096) (Fig. 4A). Moreover, *BOC* expression levels were significantly elevated with advancing cancer stages (Fig. 4B). These findings indicate a negative correlation between *BOC* expression and patient survival. Thus, *BOC* may also serve as a potential biomarker for predicting patient survival outcomes, providing valuable insights for treatment planning.

### 3.5 BOC Is Involved in Cancer Cell Migration, but Not Proliferation

**Figure 5:**
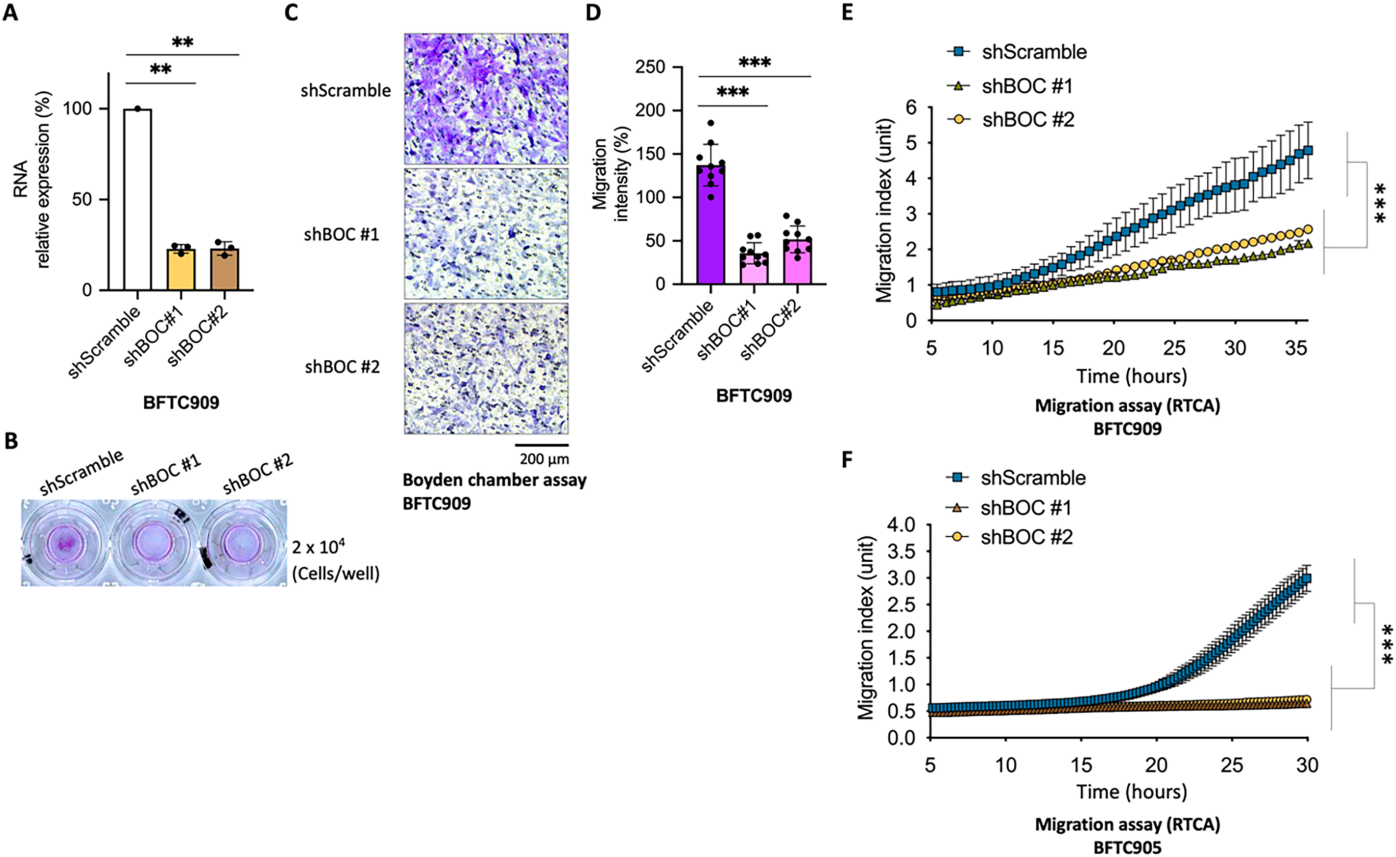
BOC silencing suppresses cell migration in urothelial carcinoma cells. (**A**) qRT-PCR confirmed efficient knockdown of BOC expression in BFTC909 cells using two independent shRNAs (**, *p* < 0.01, one-way ANOVA). (**B–D**) Representative images and quantification of Boyden chamber assays demonstrated significantly decreased migration in shBOC cells compared with controls (***, *p* < 0.001, one-way ANOVA). Scale bar: 200 µm, (**E,F**) RTCA migration assays showed impaired migration of BFTC909 cells after BOC knockdown (***, *p* < 0.001, two-way ANOVA with repeated measures and post-hoc tests).

To further elucidate the underlying mechanisms of *BOC*, a series of experiments was performed using urothelial carcinoma cells (BFTC909 cell line) to investigate its functional role. First, *BOC* expression was silenced using shRNA (shBOC) purchased from the RNAi Core Laboratory of the Academia Sinica in Taiwan. The silencing efficiency was verified with qRT-PCR primers designed by Origene (https://www.origene.com). Two different shRNA sequences (shBOC#1 and shBOC#2) were used, and silencing efficiencies of 77% and 77% were obtained (Fig. 5A). Cell proliferation and migration assays were then performed to evaluate the functional impact of *BOC* silencing.

In the cell proliferation assay using BFTC909 cells, silencing of *BOC* did not significantly affect cancer cell growth (Fig. S1). However, in the Boyden chamber migration assay, silencing *BOC* expression significantly inhibited the migration ability of the cancer cells (Fig. 5B). Specifically, shBOC#1 reduced cell migration by 74% and shBOC#2 reduced cell migration by 62% (Fig. 5C,D).

### 3.6 Enhanced Validation of BOC Silencing on Cancer Cell Migration Using RTCA

To more accurately assess the impact of *BOC* silencing on cancer cell behavior, an RTCA was used. The RTCA uses a sensitive instrument that quantifies cellular functions by monitoring electronic signal changes without manual interference. The RTCA technology continuously monitors, records, and analyzes cell activity, allowing for precise quantification of cell count and surface area over time. The results demonstrated that *BOC* silencing significantly inhibited BFTC909 cancer cell migration. The migration curves for shBOC#1 and shBOC#2 exhibited a marked reduction in migration compared to the control group (Fig. 5E).

To validate this finding, a similar migration assay was performed using another urothelial carcinoma cell line, BFTC905. The results showed that both shBOC#1 and shBOC#2 substantially inhibited cell migration (Fig. 5F). These results provide strong evidence of the role of *BOC* in promoting cell migration in urothelial carcinoma.

### 3.7 BOC Silencing Reduces Metastatic Potential in Bladder Cancer Cells

In addition to urothelial carcinoma cells, the role of *BOC* in cancer cell metastasis was examined in the T24 bladder cancer cell line. Three distinct shRNA sequences (shBOC#1, shBOC#2, and shBOC#3) were used to silence *BOC* expression, achieving silencing efficiencies of 90%, 89%, and 89%, respectively (Fig. S2A). Cell migration assays using the Boyden chamber method demonstrated a significant reduction in T24 cell migration following *BOC* silencing (Fig. S2B–D). Specifically, shBOC#1, shBOC#2, and shBOC#3 reduced cell migration by 43%, 52%, and 60%, respectively.

These findings demonstrate that *BOC* plays an important role in promoting the metastatic potential of urothelial cancers, including both UTUC and bladder cancer. However, the effect of *BOC* silencing on cell migration varied across different cell lines, with *BOC* having a more pronounced pro-metastatic effect in UTUC cells compared to bladder cancer cells.

To strengthen the clinical relevance of these findings, correlation analyses were performed using TCGA bladder urothelial carcinoma (BLCA) RNA-seq data. BOC expression showed strong positive associations with metastasis- and epithelial–mesenchymal transition (EMT)-related genes such as *MMP2, MMP9, CDH2, VIM, SNAI1*, and *TWIST1*, but not with *CDH1, TP53*, or *AKT1* (Fig. S3). In addition, BOC was positively correlated with Hedgehog pathway components, including *DHH, PTCH2, SMO, GLI1, GLI2*, and *BCL2* (Fig. S4). These results suggest that BOC expression is linked to tumor invasion and Hedgehog-related oncogenic signaling in urothelial carcinoma.

### 3.8 Association between BOC Expression and Chemotherapy Drug Sensitivity

Chemoresistance occurs frequently in patients with urothelial carcinoma. Thus, identification of predictive biomarkers for chemotherapy sensitivity and chemoresistance is important for diagnosis and response evaluation, and for the potential development of methods to enhance chemotherapy effectiveness. Thus, the relations between *BOC* expression and sensitivity to various chemotherapeutic agents were examined.

**Figure 6:**
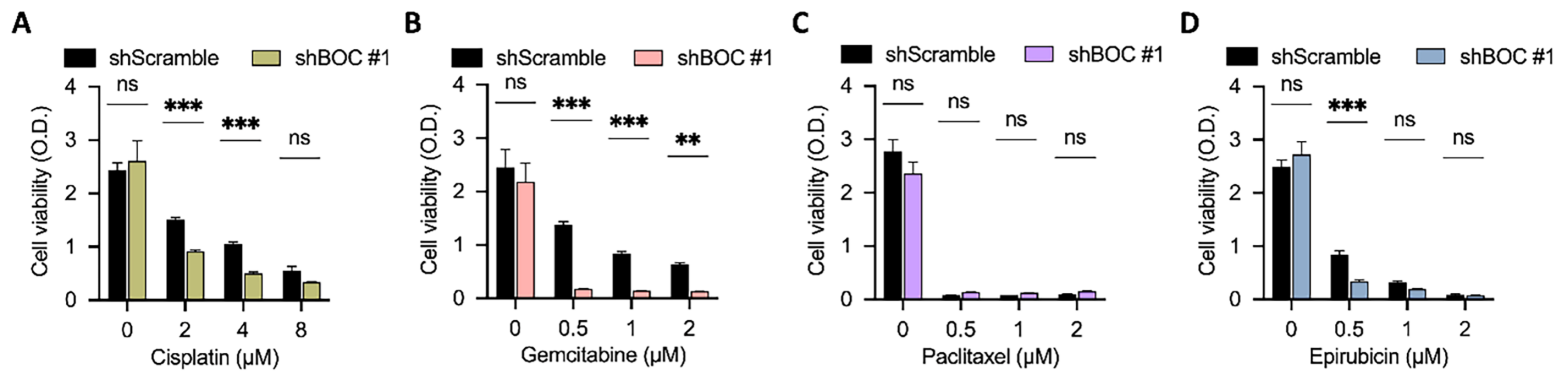
BOC silencing enhances sensitivity to cisplatin and gemcitabine in BFTC909 cells. (**A**–**D**) Cell viability assays were performed in BFTC909 cells with BOC knockdown (shBOC#1) or control (shScramble) following treatment with (**A**) cisplatin, (**B**) gemcitabine, (**C**) paclitaxel, or (**D**) epirubicin at the indicated concentrations. Data are presented as mean ± SEM. All statistics were determined using the one-way ANOVA. **, *p* < 0.01; ***, *p* < 0.001; ns, not significant.

The results showed that in BFTC909 cells, *BOC* silencing (shBOC) in combination with cisplatin (Fig. 6A) had a synergistic inhibitory effect on cell growth compared to the shScramble control group. *BOC* silencing also significantly increased sensitivity to gemcitabine (Fig. 6B). The effect on paclitaxel sensitivity was less clear (Fig. 6C). For epirubicin, the effect was significant at lower concentrations, while at higher doses the viability of BOC-silenced cells was not different than that of the control group (Fig. 6D).

**Figure 7:**
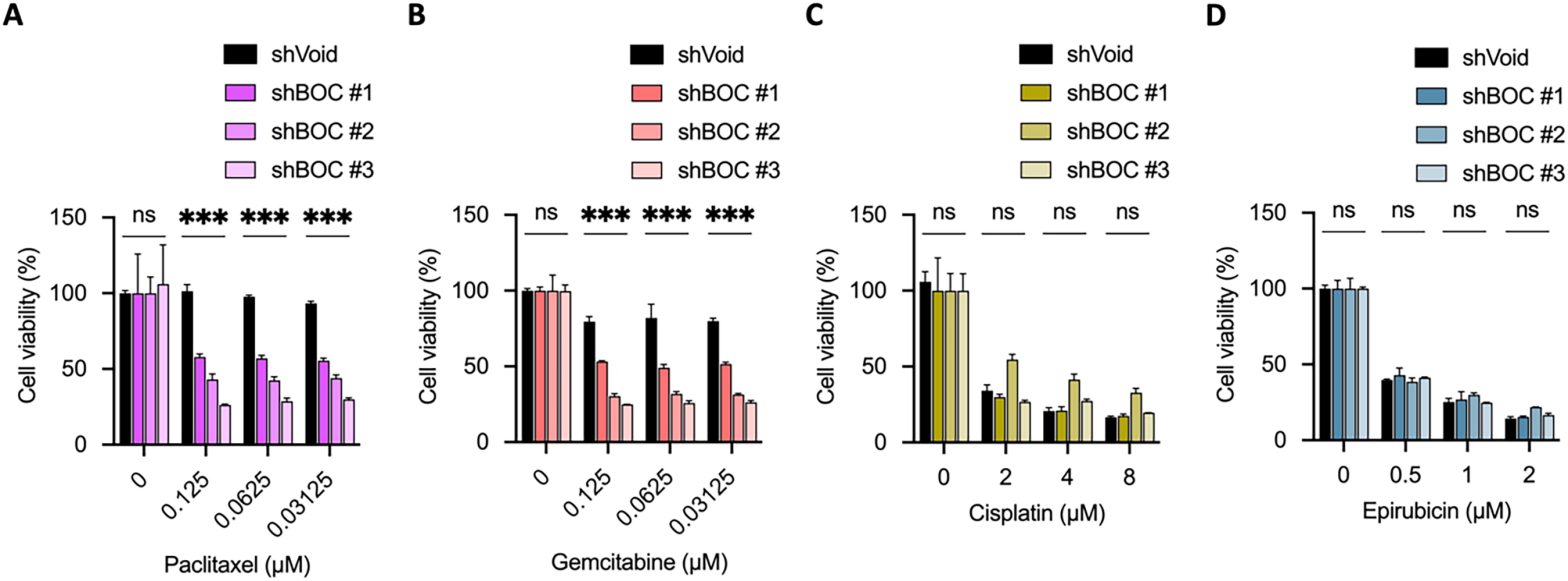
BOC silencing enhances sensitivity to paclitaxel and gemcitabine in T24 cells. (**A**,**B**) BOC knockdown significantly reduced cell viability in response to paclitaxel and gemcitabine. (**C**) Cell viability after cisplatin treatment in BOC knockdown (shBOC#1–3) and control (shVoid) cells showed no significant difference. (**D**) Epirubicin treatment showed no significant difference between shBOC and control cells. Data are presented as mean ± SEM. All statistics were determined using the one-way ANOVA. ***, *p* < 0.001; ns, not significant.

Further analysis using the T24 bladder cancer cell line demonstrated that *BOC* silencing enhanced the efficacy of low-dose paclitaxel and gemcitabine (Fig. 7A,B). However, no synergistic effects were observed at higher doses of these drugs (Fig. S5). In contrast, no association was found between *BOC* silencing and cell growth inhibition with cisplatin or epirubicin in T24 cells (Fig. 7C,D). Additionally, in the UTUC cell line BFTC905, *BOC* silencing did not significantly enhance drug response (Fig. S6). This intercellular variability demonstrates that the impact of BOC on drug sensitivity is model-specific, likely reflecting intrinsic molecular heterogeneity among cell lines.

To further investigate the molecular mechanism underlying the differential chemosensitivity among cell lines, we performed RNA-seq analysis in BFTC909 cells following *BOC* knockdown (shBOC) compared with control cells (shScramble). The expression levels of Hedgehog pathway–related genes, including *PTCH1, SMO, SUFU, GLI1, Myc, BCL2, FOXM1*, and *VEGFA*, were examined. *BOC* knockdown resulted in a significant reduction of *VEGFA* expression (*p* = 0.046). Notably, *SMO* expression also showed a marked downward trend, with transcript levels strongly suppressed in shBOC cells despite a marginal *p-*value (*p* = 0.098). Other Hedgehog-related genes showed no significant changes between groups (Fig. S7). Because *SMO* and *VEGFA* are both known to correlate positively with chemotherapeutic efficacy, these findings suggest that *BOC* may influence chemosensitivity through partial regulation of Hedgehog and *VEGFA*-mediated angiogenic signaling. Such variation across cell lines or tumor tissues may also reflect differences in the activity of these pathways, which should be further investigated in future studies.

### 3.9 Identification and Expression Analysis of Methylation Sites in the BOC Gene

Given the significant difference in *BOC* gene expression between LVI(+) and LVI(−) tissues, methylation analysis of the *BOC* gene was performed to identify specific methylation sites that may influence differential *BOC* expression, and thus provide potential targets for future drug development.

**Figure 8:**
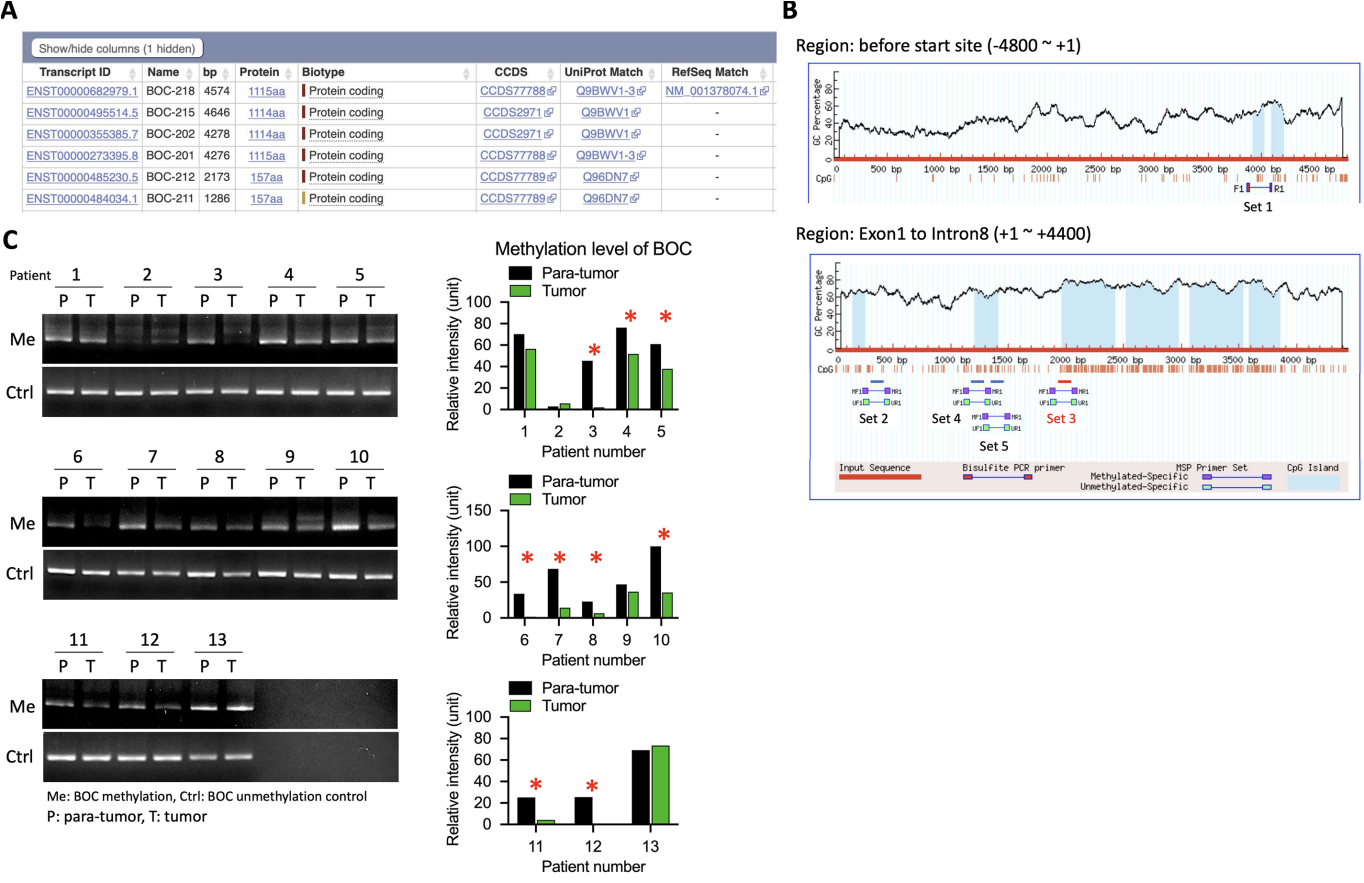
Increased BOC promoter methylation was observed in tumor samples compared with para-tumor tissues in UTUC patients. (**A**) List of BOC transcript isoforms with annotated transcript IDs. (**B**) CpG island prediction and primer design in the BOC promoter and exon 1–intron 8 regions. (**C**) Methylation-specific PCR (MSP) analysis of BOC in paired para-tumor (P) and tumor (T) tissues from 13 UTUC patients, with representative bands for methylated (Me) and control (Ctrl) lanes. Quantification of relative methylation levels is shown on the right. *, *p* < 0.05; two-way ANOVA.

Transcript sequence information for *BOC* from the Ensembl database (ENST00000682979.1) was used (Fig. 8A), and locations of CpG islands within the *BOC* gene were identified using MethPrimer (Fig. 8B). The MSP primer prediction tool was then used to determine suitable regions within the *BOC* gene for detecting methylation levels. The analysis predicted 5 methylation sites within the −5000 bp to +5000 bp region of *BOC* (Fig. 8B). Corresponding methylation-specific primer sets and sequences were designed for these sites.

To assess the impact of these methylation sites, methylation-detection primers were synthesized, and methylation-specific PCR assays on DNA samples from 13 additional UTUC patient tumor and para-tumor tissues were performed. The results showed that among the 5 predicted methylation sites, Set 3, located near the +2000 bp region, was the optimal site for methylation detection (Fig. 8B). In the 13 UTUC tumor tissues, 9 exhibited significantly lower methylation levels at this site compared to adjacent normal tissues, indicating that Set 3 is a reliable predictor of *BOC* methylation status (Fig. 8C).

### 3.10 Differential Expression of BOC in Low- and High-Grade Bladder Urothelial Carcinoma

To further investigate the expression of the *BOC* gene across different tumor grades, we analyzed IHC staining data from 11 bladder urothelial carcinoma tumor samples in the Human Protein Atlas (4 low-grade and 7 high-grade tumor samples). In normal tissue, *BOC* expression was observed in the cytoplasm with enhanced expression in the nucleus (Fig. S8A). In the tumor samples, *BOC* expression was visualized using red to denote areas of increased expression after background correction, enabling quantitative analysis (Fig. S8B).

**Figure 9:**
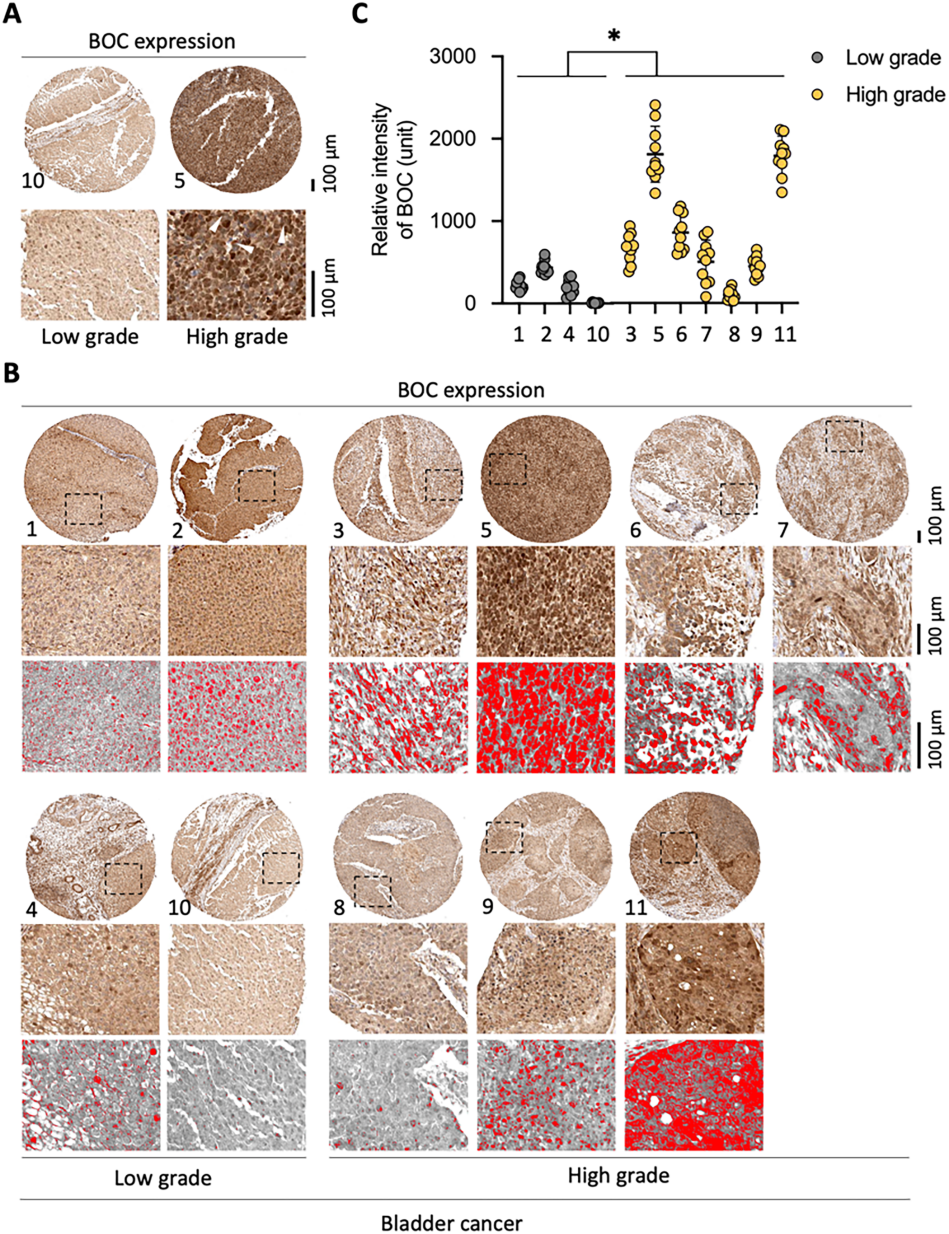
High BOC expression is associated with high-grade bladder cancer. (**A**) Representative immunohistochemistry (IHC) staining of BOC in low-grade and high-grade bladder cancer tissues. (**B**) Additional representative IHC staining panels with digital quantification masks (red). (**C**) Quantitative analysis of BOC staining intensity shows significantly higher expression in high-grade compared with low-grade tumors. *, *p* < 0.05; two-way ANOVA. All scale bars: 100 µm.

Ten random locations within each sample were selected to assess relative expression intensity. The results showed differential *BOC* expression between low- and high-grade tumor tissues. Notably, in some high-grade tumor sections (e.g., sample #5), elevated *BOC* expression was observed in both the cytoplasm and nucleus, while in low-grade tumors (e.g., sample #10), *BOC* expression resembled that seen in normal tissue (Fig. 9A). The expression patterns of all 11 samples, with selected areas magnified to highlight *BOC* expression levels in red are shown in Fig. 9B, and the quantified relative expression intensity for each sample is shown in Fig. 9C. Statistical analysis revealed that *BOC* expression was significantly higher in high-grade compared to low-grade tumors (*p* < 0.001, Fig. 9C), suggesting that *BOC* may be associated with tumor malignancy and could serve as a biomarker for assessing tumor grade.

Notably, enhanced nuclear expression of *BOC* was observed in some low-grade (sample #2) and high-grade tumors (samples #3, #5, and #6), indicating a possible role for nuclear *BOC* in tumor aggressiveness, which may reflect invasive tumor characteristics.

### 3.11 Association of BOC Expression with Clinicopathological Features in UTUC

We further examined the clinical relevance of BOC expression in upper tract urothelial carcinoma (UTUC). Relative BOC expression levels were quantified by qRT-PCR in 48 UTUC tumor samples. BOC expression was significantly higher in stage III tumors compared with stage I tumors (*p* = 0.0405, unpaired Student’s *t*-test; Fig. S9). In addition, metastatic tumors exhibited significantly higher BOC expression than non-metastatic tumors (n = 48; 42 non-metastatic and 6 metastatic; *p* = 0.0255, unpaired Student’s *t*-test; Fig. S10).

These results are consistent with the *in vitro* functional data, supporting the role of BOC in promoting tumor aggressiveness and metastatic potential in urothelial carcinoma. Collectively, these clinical observations provide supportive evidence for a potential link between high BOC expression and advanced disease progression.

## 4 Discussion

The results of this study identified *BOC* as a marker for LVI and cancer progression in UTUC. RNA sequencing of tissue specimens from 10 patients with UTUC, followed by validation in 2 independent cohorts totaling 74 patients, confirmed significantly higher *BOC* expression in LVI(+) UTUC. Functional analysis showed that *BOC* silencing inhibits cancer cell migration, but does not affect proliferation. Chemotherapy sensitivity tests revealed that *BOC* silencing enhances sensitivity to cisplatin and gemcitabine in UTUC cells, though the effect varies depending on cell type and drug dosage. Methylation analysis identified a key regulatory site controlling *BOC* expression, while IHC staining demonstrated higher BOC levels in high-grade bladder cancer, suggesting its role in tumor aggressiveness. Consistent with these findings, transcriptomic and clinical analyses further supported that BOC expression is associated with epithelial–mesenchymal transition, metastatic potential, and advanced tumor stage. These results suggest that BOC may promote tumor progression through EMT and Hedgehog-related signaling pathways. Collectively, these findings suggest that *BOC* may serve as a potential biomarker candidate for LVI, metastasis, and chemotherapy response in patients with UTUC, although further validation in larger, multi-center cohorts is warranted.

The *BOC* gene encodes a member of the immunoglobulin/fibronectin III repeat family of receptor-like proteins that binds to Sonic hedgehog and regulates downstream signaling [24,31,32]. Increased BOC expression has been identified in medulloblastoma and uterine mesenchymal tumors, and is a prognostic marker in renal clear cell carcinoma and ovarian cystadenocarcinoma [24,33–36]. BOC has also been shown to play a role in myogenic differentiation, synaptic development, and axon guidance [24,37–39]. Mathew et al. [40] studied the role of BOC in pancreatic cancer. In pancreatic cancer, tumor expression of hedgehog (HH) ligands signals fibroblasts, which promotes tumor survival and growth. The study showed that the HH coreceptors BOC, GAS1, and CDON are expressed in cancer-associated fibroblasts, and that deletion of GAS1 and BOC decreased HH signaling, and deletion of all 3 almost completely stopped HH signaling and failure of tumorigenesis and angiogenesis. Pedrosa et al. [41] studied brain metastases in women with estrogen receptor ER-negative breast cancer, and reported that *BOC*, as well as the genes *SPOCK2* and *GJD3*, were overexpressed in primary breast tumors that developed brain metastasis.

Our results showed that BOC was associated with LVI in UTUC. LVI is associated with more advanced stages of many malignancies and is associated with a poor prognosis because once malignant cells have entered the vascular or lymphatic system, there is a route for dissemination to distant sites [42–45]. LVI is more common in certain types of cancer, such as breast cancer, colon cancer, and endometrial cancer, and depending on the malignancy, patients with LVI may require adjuvant chemotherapy or radiation therapy as part of their treatment [46–50]. LVI can occur as a result of tumor-specific processes, as well as normal immunological processes that are “used” by malignant cells to enter the lymphatic or vascular system [42,51,52]. Smoothened is a protein encoded by the *SMO* gene, and is part of the HH signaling pathway, which regulates cell growth, migration, invasion, and stem cells in malignancies [53–55]. In a recent study, Wang et al. [56] studied BOC expression in relation to glioblastoma cells. The overall results of their study showed that BOC is aberrantly overexpressed in patients with glioma and promotes glioma development. Their experiments showed that BOC upregulates the expression of SMO (and EGFR, HRAS, and MRAS), which activate HH and RAS signaling pathways and thus facilitate the proliferation, invasion, and migration of glioma cells. Building upon these mechanistic insights, our supplementary analyses further support a potential role of BOC in modulating Hedgehog-related signaling and angiogenic activity in urothelial carcinoma. Specifically, transcriptomic and RNA-seq data indicated that reduced BOC expression was accompanied by decreased SMO and VEGFA activity, consistent with a role for BOC in HH pathway–mediated tumor regulation, although further studies are needed to clarify the underlying regulatory mechanisms. In addition, in a study that aligns with our research, Nedjadi et al. [57] showed that high expression of the HH pathway was significantly associated with increased lymph node metastasis in urothelial bladder cancer, which aligns with our findings and further supports the mechanistic association proposed in this study.

Because UTUC is typically diagnosed at an advanced stage, determining sensitivity to different chemotherapy drugs is important for treatment success [58–60]. Li et al. [61] developed a platform of patient-derived UTUC organoids for drug screening, and using the platform to examine gemcitabine resistance, the results indicated that treatment with gemcitabine results in the upregulation of pathways associated with cellular resistance. With respect to cisplatin resistance in UTUC, Yu et al. [62] used cisplatin-resistant and cisplatin-sensitive human urothelial carcinoma cell lines and reported that resistance was not related to the intracellular platinum concentration. Instead, the study showed that the overexpression of anti-apoptotic Bcl-2, anti-oxidative heme oxygenase-1 (HO-1), and cell cycle regulator p16INK4 was responsible for cisplatin resistance. Notably, cisplatin has been a cornerstone of chemotherapy for many decades, despite its measurable toxicity [63]. As such, a great deal of research has gone into understanding how resistance to cisplatin develops, and how to overcome/reduce resistance to treatment [63].

The findings of the current study have many clinical implications for the treatment of UTUC. For example, a systematic review and meta-analysis by Ku et al. [64] showed that LVI was associated with increased mortality in patients with UTUC. Thus, establishing the presence of LVI at diagnosis can guide the application of more aggressive initial treatments and potentially improve outcomes. Similarly, a systematic review and meta-analysis by Sharma et al. [65] reported that tumor grade and stage, the presence of LVI, lymph node metastasis, and a number of other factors were associated with poorer OS, recurrence-free survival, and cancer-specific survival in patients with UTUC.

Given the overall poor prognosis of advanced urothelial carcinoma, increasing research efforts have focused on the development of novel systemic treatment strategies, including targeted therapies, immunotherapy, and antibody–drug conjugates (ADCs). Recent advances summarized by Wang et al. indicate that FDA-approved ADCs, such as enfortumab vedotin and sacituzumab govitecan, have expanded therapeutic options for advanced urothelial carcinoma [66]. However, clinical responses remain heterogeneous, and reliable predictive biomarkers for treatment efficacy and resistance are still lacking [67–70]. These findings underscore the clinical need to identify novel molecular markers associated with tumor aggressiveness and therapeutic sensitivity, which may include BOC.

### 4.1 Strengths and Limitations

Strengths of this study include multi-stage validation, integration of RNA-seq and TCGA data, and functional studies in urothelial carcinoma cell lines, which collectively demonstrate the role of BOC in cancer cell migration and its potential to modulate chemosensitivity to cisplatin and gemcitabine. Furthermore, the identification of specific BOC methylation sites and their elevated expression in high-grade tumors underscores their relevance in tumor progression and opens avenues for exploring methylation-based treatments. However, limitations include a moderate sample size, lack of long-term survival data, cell line-dependent chemoresistance findings, and the unvalidated functional relevance of the methylation sites. The variability among cell models suggests that the chemosensitizing effects of BOC may depend on specific molecular or cellular contexts, which should be further clarified in future studies. In addition, the current methylation analysis indicates a potential association between methylation status and BOC expression, but further studies are needed to determine whether this relationship reflects a direct regulatory mechanism contributing to its oncogenic activity. Future work using next-generation bisulfite sequencing or other high-resolution methylome profiling should also aim to verify the functional significance of the identified methylation sites and their potential regulatory roles in BOC expression. Furthermore, larger, prospectively designed studies with long-term follow-up, *in vivo* model validation, and deeper exploration of the underlying molecular mechanisms of BOC effects in UTUC, such as its involvement in Hedgehog signaling and cell adhesion pathways, are warranted.

### 4.2 Conclusions

This study provides preliminary evidence that BOC may serve as a potential biomarker for LVI and drug response in UTUC. BOC expression was significantly higher in LVI(+) patients, and its silencing inhibited cancer cell migration without affecting proliferation. Additionally, BOC silencing enhanced sensitivity to cisplatin and gemcitabine, although the effects varied by drug and cell type. Methylation analysis revealed key regulatory sites that may control BOC expression, and IHC confirmed higher BOC levels in high-grade bladder cancer, linking its expression to tumor progression and metastasis. While these findings support the potential role of BOC in UTUC progression and chemosensitivity, further validation in larger, multi-center, and prospective cohorts is warranted to confirm its clinical relevance. Future research should also investigate the mechanistic pathways *in vivo* and evaluate the therapeutic potential of targeting BOC and its methylation regulation.

## Supplementary Materials



## Data Availability

The original contributions presented in this study are included in the article/supplementary material. Further inquiries can be directed to the corresponding authors.

## Supplementary Materials

The supplementary material is available online at https://www.techscience.com/doi/10.32604/or.2026.070837/s1.
