# Supplementary material for: Pre-Treatment BOC Expression as an Indicator of Lymphovascular Invasion and In Vitro Chemotherapeutic Response in Upper Tract Urothelial Carcinoma
Source: Oncol Res. 2026 Mar 23;34(4):19. doi: 10.32604/or.2026.070837 (PMC13040342; doi:10.32604/or.2026.070837)
Supplement: Supplementary file 1 [file OncolRes-34-70837-s001.zip › TSP_OR_70837-s001.docx]

Supplementary Figures


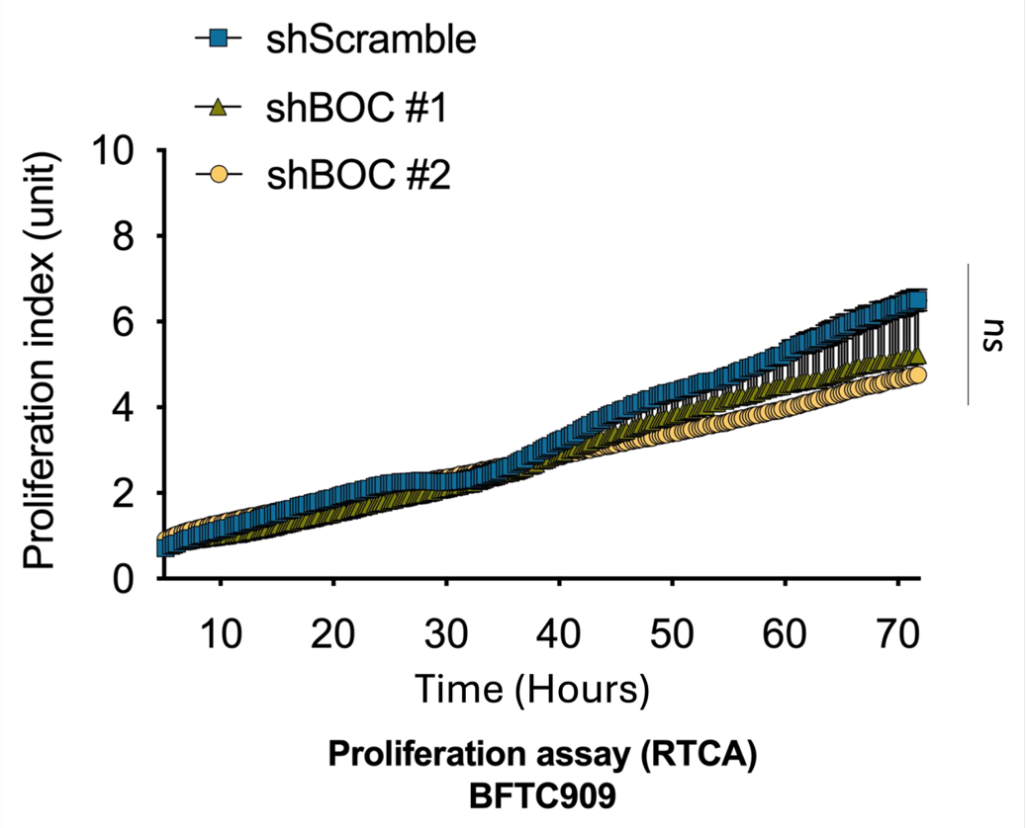


**Figure S1:** BOC knockdown does not affect the proliferation of BFTC909 cells. Real-time cell analysis (RTCA) proliferation assays of BFTC909 cells transduced with shScramble or two independent shRNAs targeting BOC (shBOC #1 and shBOC #2). No significant difference in proliferation index was observed. ns, not significant; two-way ANOVA with repeated measures.


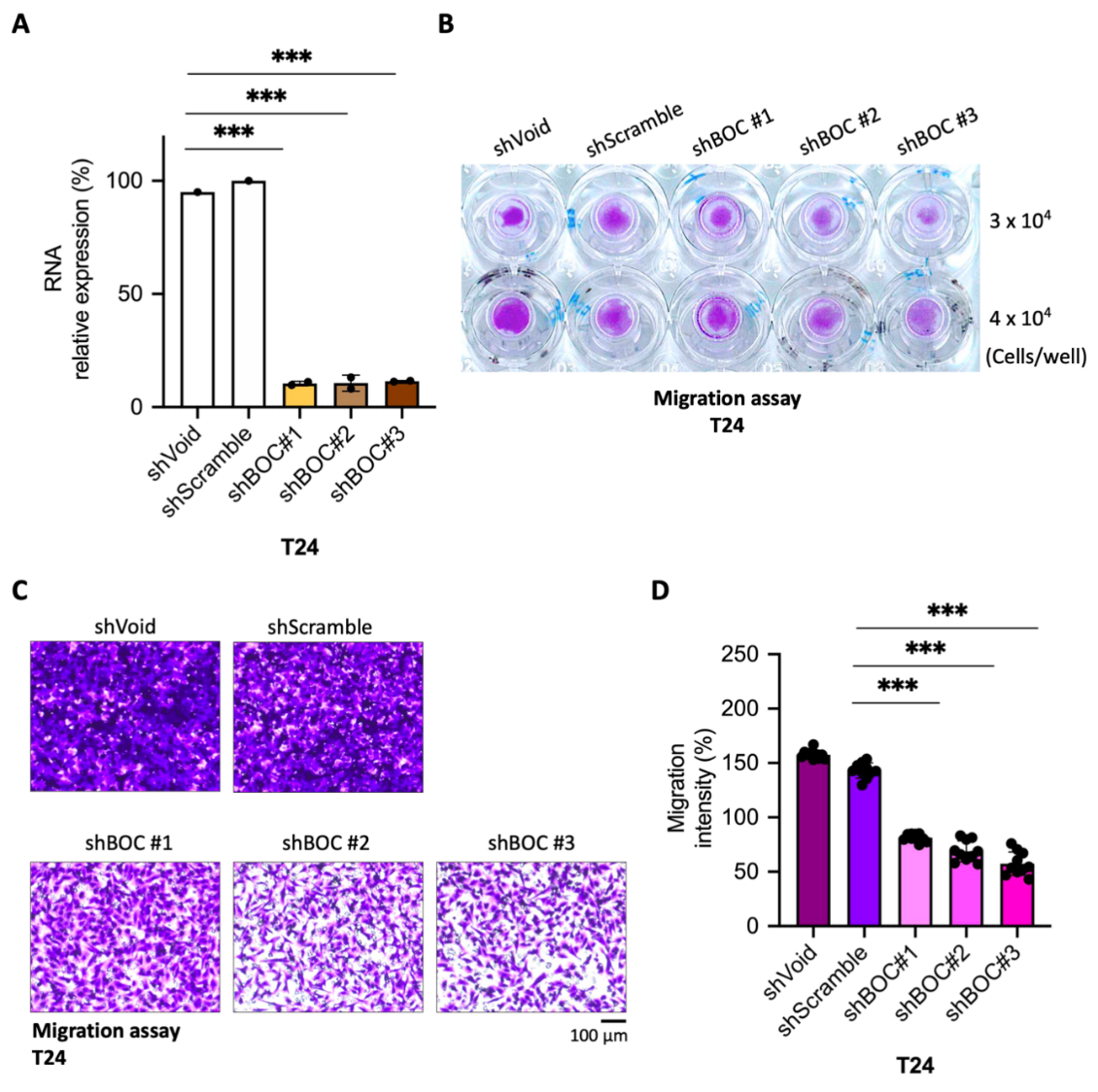


**Figure S2:** BOC knockdown suppresses the migration of T24 bladder cancer cells. (A) qRT-PCR confirmed efficient knockdown of BOC expression in T24 cells using three independent shRNAs (***, *p* < 0.001; one-way ANOVA). (B) Boyden chamber images of migration assay in T24 cells seeded at 3 × 10^4^ or 4 × 10^4^ cells/well after BOC knockdown. (C) Representative Boyden chamber assay images showing reduced migration of shBOC#1–3 compared with controls. Scale bars: 100 µm. (D) Quantification of migration intensity indicated significant suppression of cell migration in shBOC knockdown groups compared with shVoid and shScramble controls (***, *p* < 0.001; one-way ANOVA).


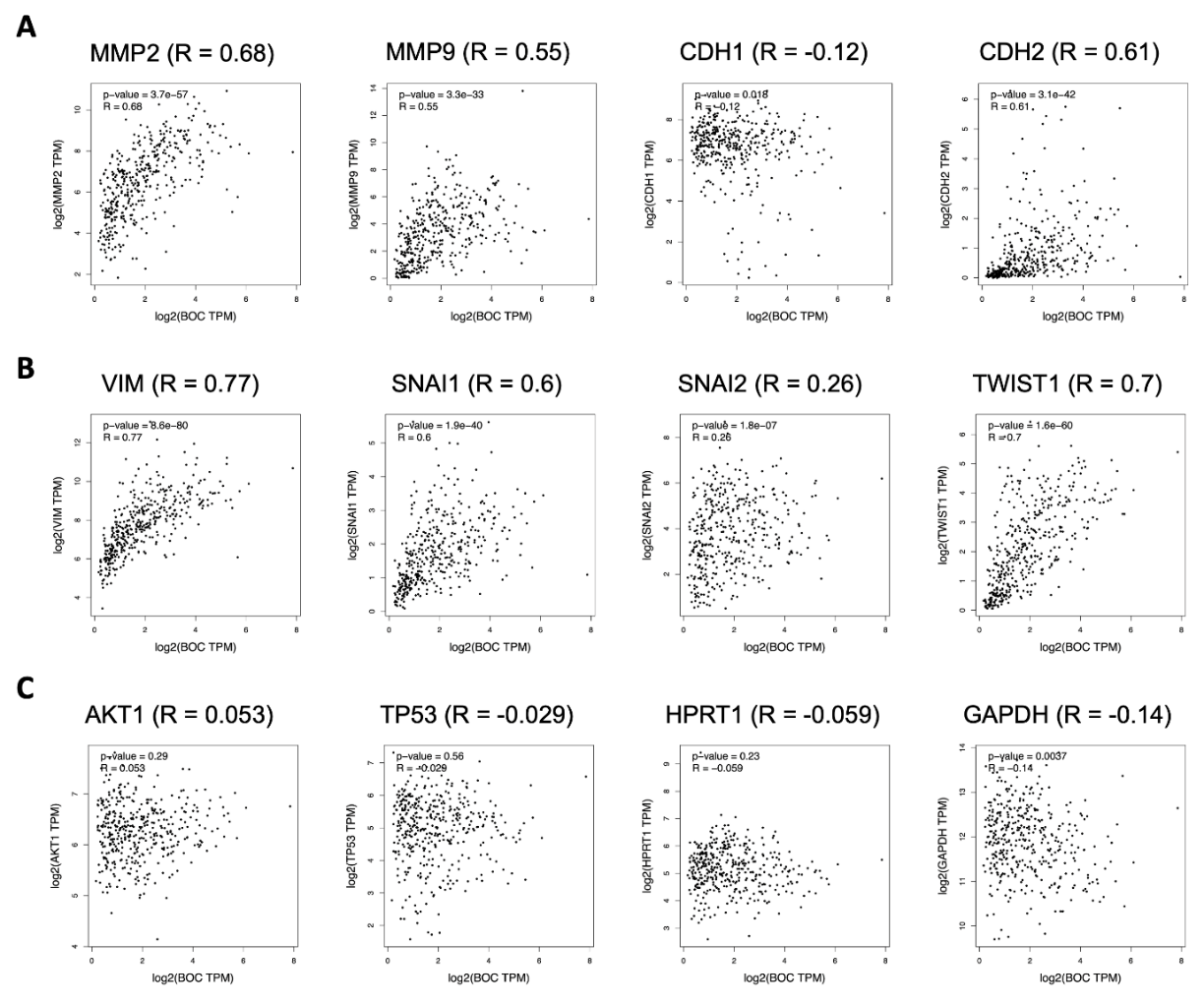


**Figure S3:** Correlation between BOC expression and metastasis-associated genes in the TCGA BLCA dataset. Correlation analyses were performed using TCGA BLCA RNA-seq data to examine the association between BOC expression and known metastasis-related genes. (A) BOC expression was positively correlated with MMP2, MMP9, CDH2 and showed no significant association with CDH1. (B) BOC expression was significantly associated with mesenchymal/EMT markers, including VIM, SNAI1, SNAI2, and TWIST1. (C) No significant associations were observed between BOC expression and AKT1, TP53, HPRT1, and GAPDH. All statistics were determined using Spearman correlation analysis.


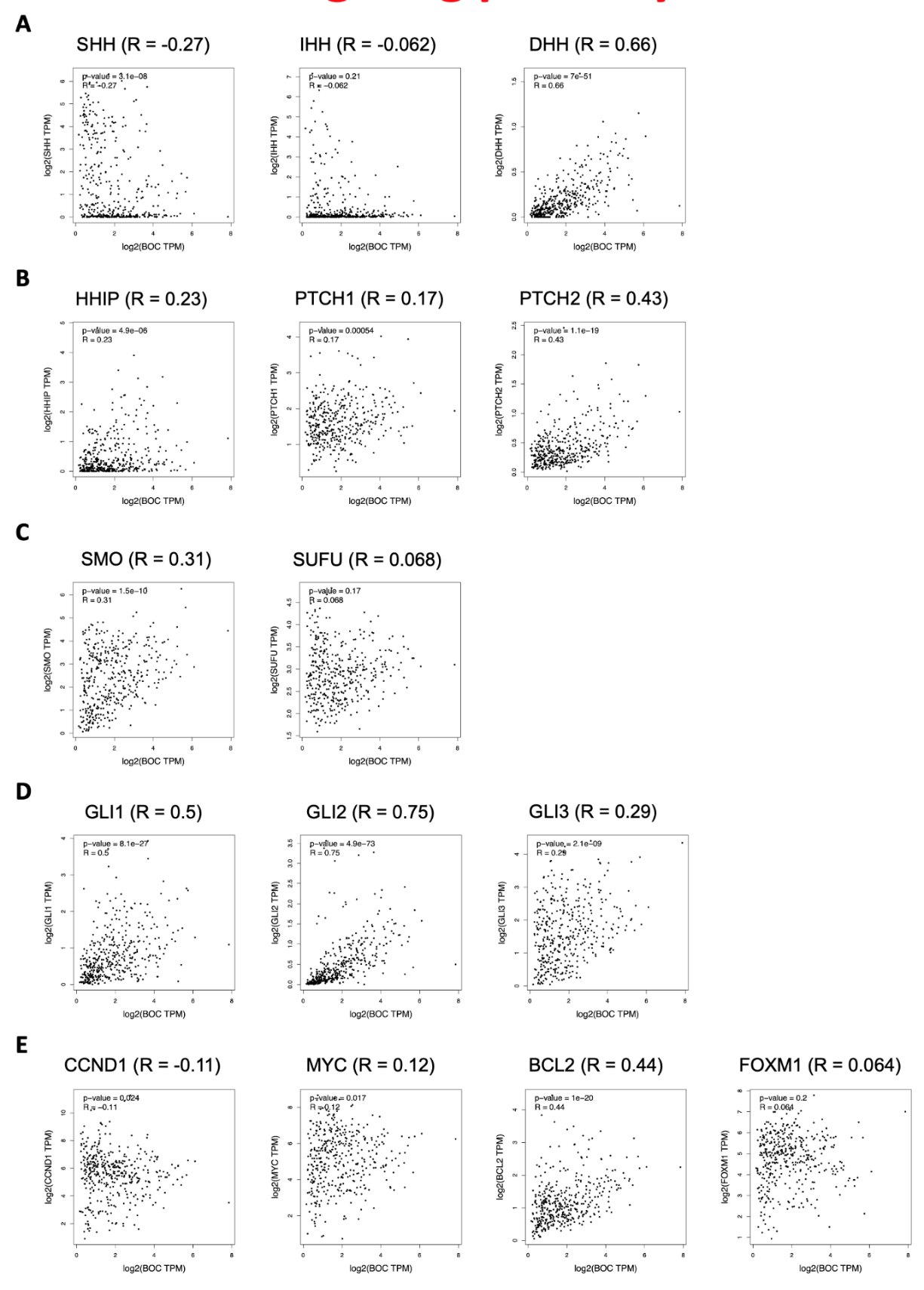


**Figure S4:** Correlation between BOC expression and Hedgehog pathway genes in the TCGA BLCA dataset. Correlation analyses were performed using TCGA BLCA RNA-seq data to examine the association between BOC expression and key components of the Hedgehog signaling pathway. (A) BOC expression showed negative or weak correlations with SHH and IHH, but a strong positive correlation with DHH. (B) Among Hedgehog ligand modulators and receptors, BOC expression was positively correlated with HHIP, PTCH1, and PTCH2. (C) Among pathway mediators, BOC expression exhibited a moderate positive correlation with SMO, while no significant association was observed with SUFU. (D) Strong positive correlations were observed between BOC expression and downstream transcription factors GLI1 and GLI2, with a weaker positive association with GLI3. (E) BOC expression also showed a positive association with downstream oncogenic targets, including BCL2, while correlations with CCND1, MYC, and FOXM1 were weak or nonsignificant. All statistics were determined using Spearman correlation analysis.


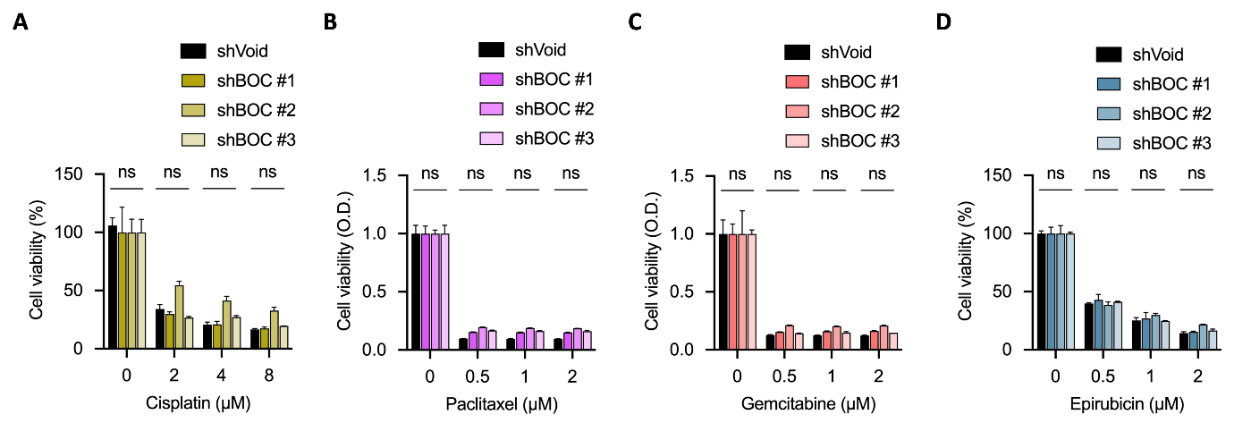


**Figure S5:** Effect of BOC knockdown on drug response in T24 bladder cancer cells. (A–D) Cell viability assays were performed in T24 cells with BOC knockdown (shBOC#1–3) or control (shVoid) following treatment with (A) cisplatin, (B) paclitaxel, (C) gemcitabine, or (D) epirubicin at the indicated concentrations. Data are presented as mean ± SEM. All statistics were determined using one-way ANOVA. ns, not significant.


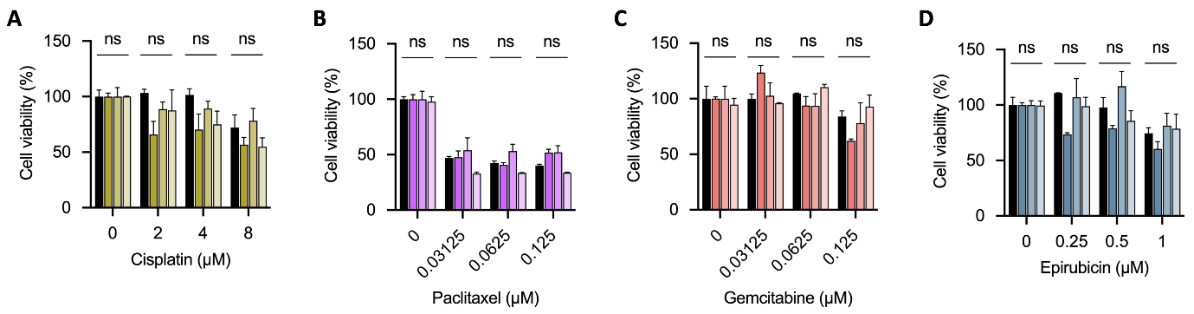


**Figure S6:** Effect of BOC knockdown on drug response in BFTC905 bladder cancer cells. (A–D) Cell viability assays were performed in BFTC905 cells with BOC knockdown (shBOC#1–3) or control (shVoid) following treatment with (A) cisplatin, (B) paclitaxel, (C) gemcitabine, or (D) epirubicin at the indicated concentrations. Data are presented as mean ± SEM. All statistics were determined using one-way ANOVA. ns, not significant.


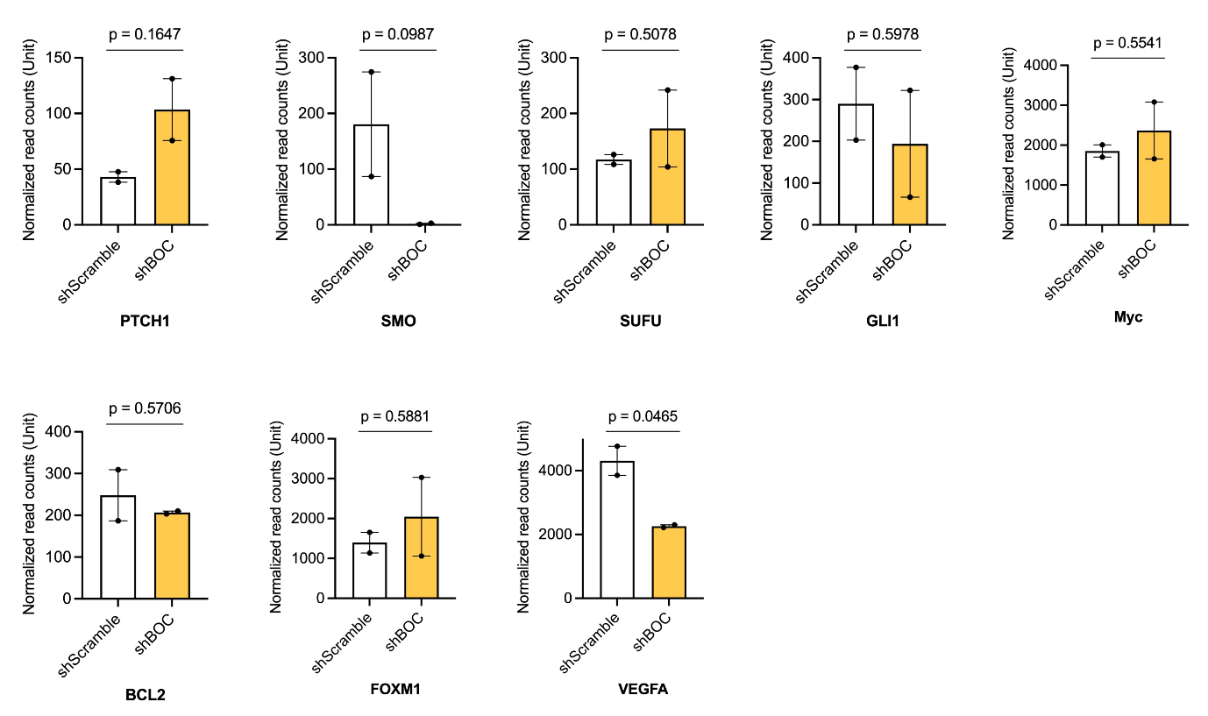


**Figure S7:** Association between BOC knockdown and Hedgehog pathway gene expression. RNA-seq analysis was performed in BFTC909 cells following BOC knockdown (shBOC) compared with control cells (shScramble). Normalized read counts of Hedgehog pathway–related genes, including PTCH1, SMO, SUFU, GLI1, Myc, BCL2, FOXM1, and VEGFA, were examined. BOC knockdown resulted in a significant reduction of VEGFA expression (*p* = 0.0465), whereas other Hedgehog pathway components showed no significant differences between groups (all *p* > 0.05, unpaired Student’s *t*-test). Bars represent the mean ± SEM.


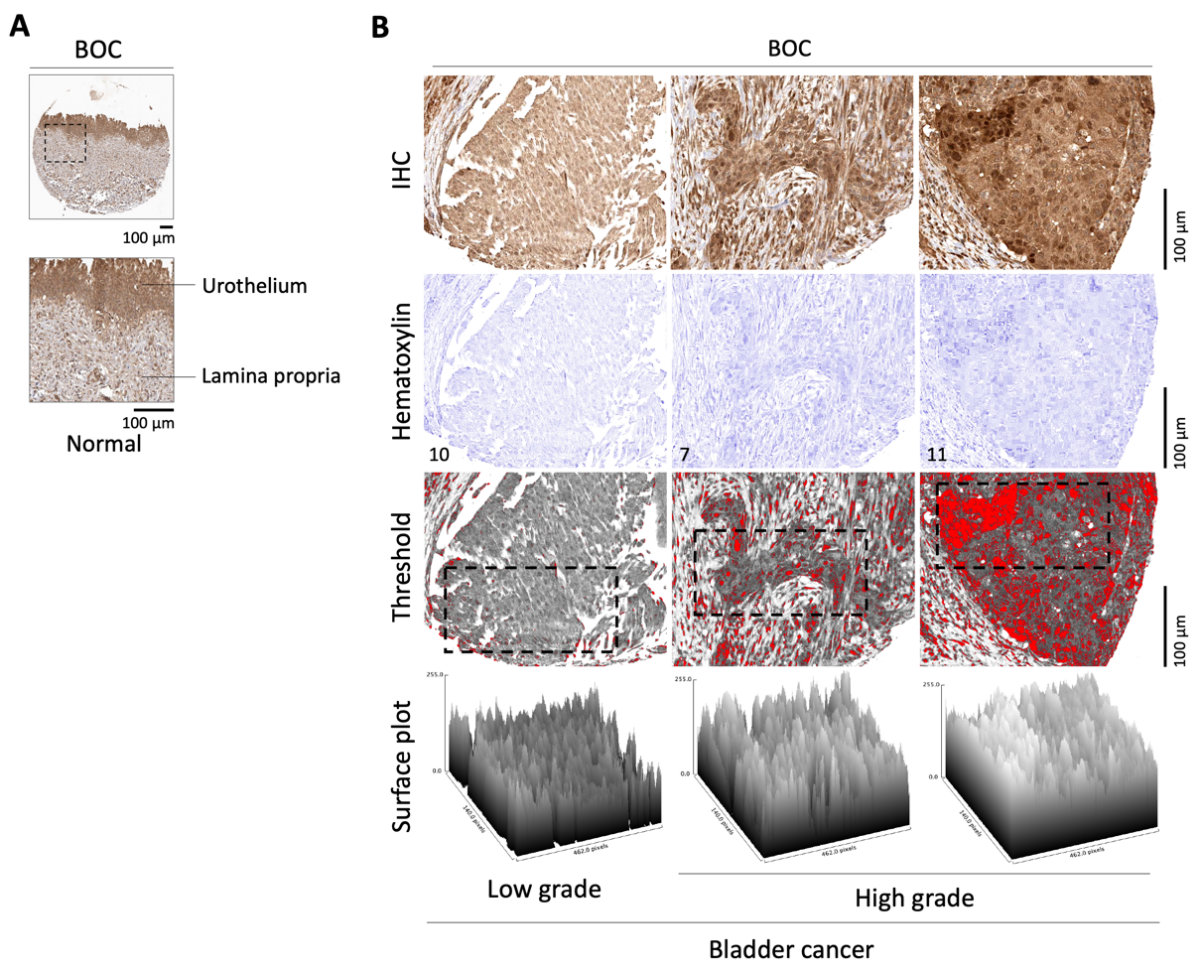


**Figure S8:** Representative images of BOC IHC quantification in bladder cancer tissues. (A) Immunohistochemistry (IHC) image from the Human Protein Atlas showing BOC localization in the urothelium and lamina propria of normal bladder tissue. (B) Representative IHC images of BOC in bladder cancer tissues. The original IHC images were processed using ImageJ IHC Profiler to generate hematoxylin-enhanced visualizations, threshold segmentation (red), and 3D surface plots for comparison between low-grade and high-grade tumors. All scale bar: 100 µm.


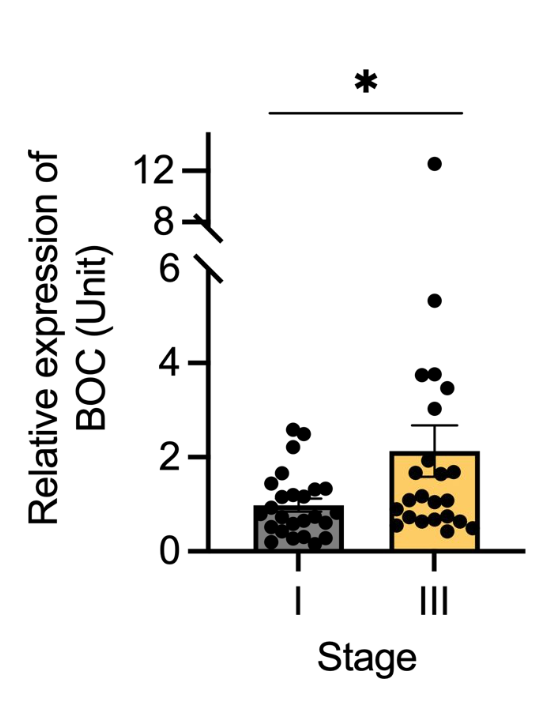


**Figure S9:** Association between BOC expression and stage in UTUC patients. Relative expression levels of BOC were quantified by qRT-PCR in UTUC patient samples and compared between stage I (n = 25) and stage III (n = 23) tumors. Each dot represents an individual patient sample, and bars indicate the mean ± SEM. BOC expression was significantly higher in stage III tumors (*p* = 0.0405, unpaired Student’s *t*-test). *, *p* < 0.05.


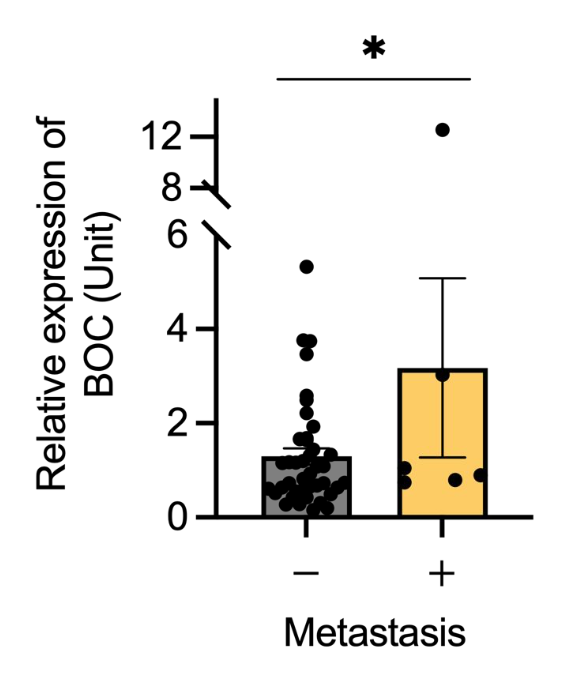


**Figure S10:** Association between BOC expression and metastasis in UTUC patients. Relative expression levels of BOC were quantified by qRT-PCR in UTUC patient samples and compared between tumors without metastasis (n = 42) and with metastasis (n = 6). Each dot represents an individual patient sample, and bars indicate the mean ± SEM. BOC expression was significantly higher in metastatic tumors (*p* = 0.0255, unpaired Student’s *t*-test). *, *p* < 0.05.
