# Supplementary figures and images for: Pre-Treatment BOC Expression as an Indicator of Lymphovascular Invasion and In Vitro Chemotherapeutic Response in Upper Tract Urothelial Carcinoma
Source: Oncol Res. 2026 Mar 23;34(4):19. doi: 10.32604/or.2026.070837 (PMC13040342; doi:10.32604/or.2026.070837)

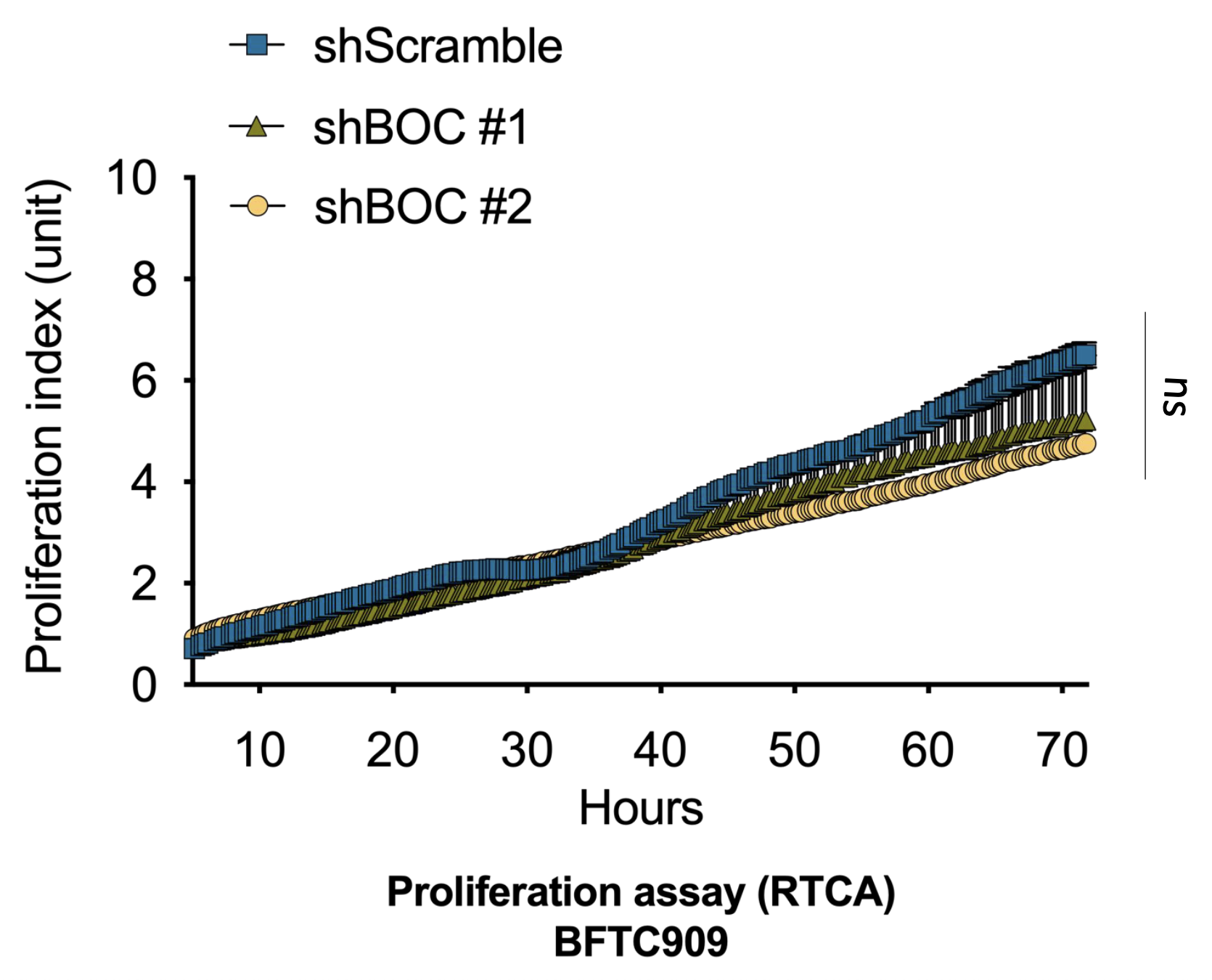

Supplement: Supplementary file 1 [file OncolRes-34-70837-s001.zip › TSP_OR_70837-s001-figures/Fig S1.tiff]

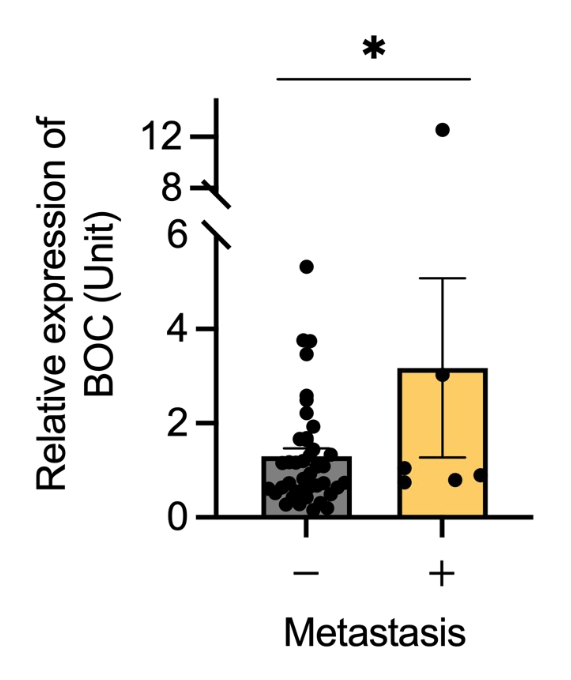

Supplement: Supplementary file 1 [file OncolRes-34-70837-s001.zip › TSP_OR_70837-s001-figures/Fig S10.tiff]

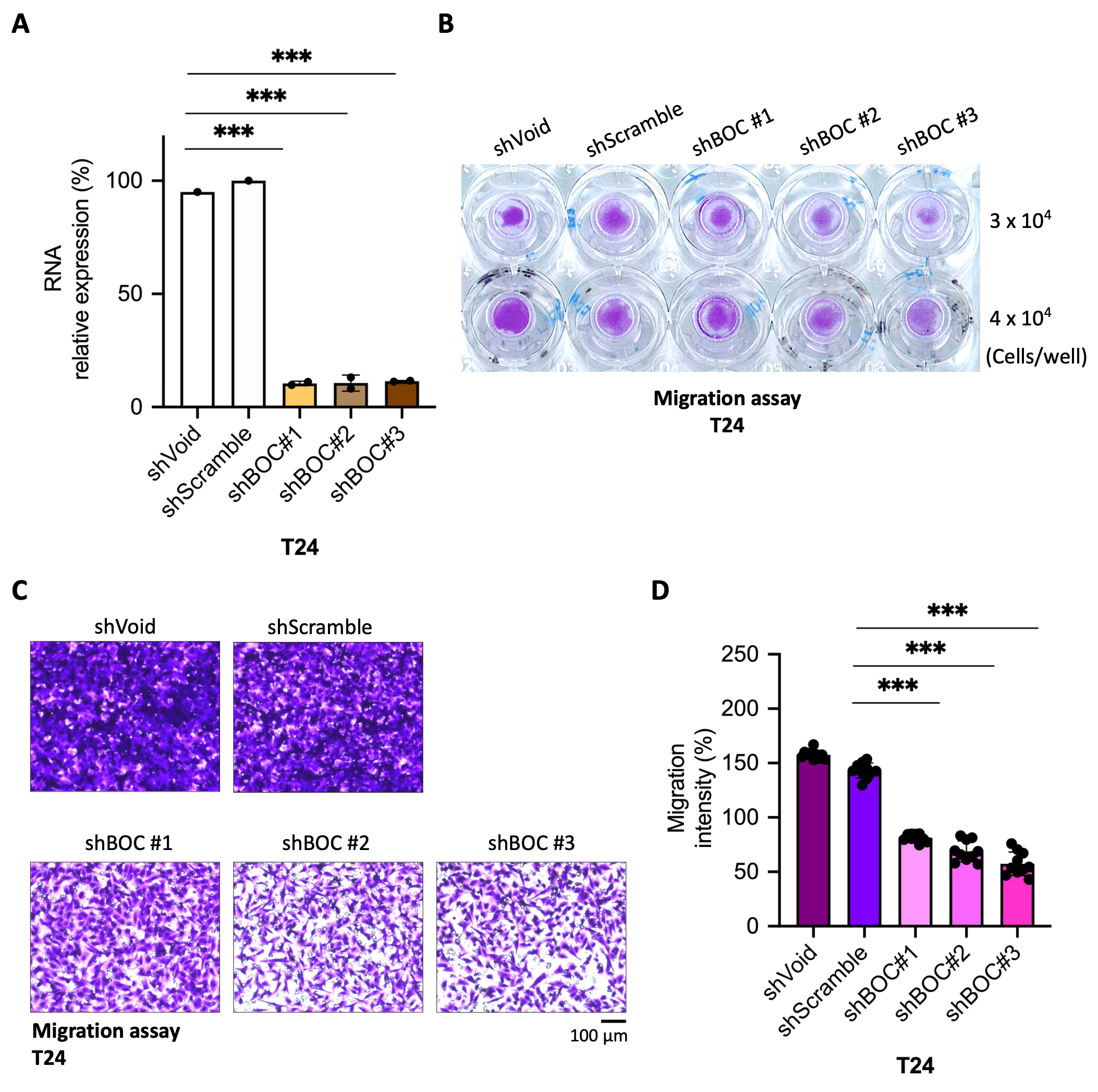

Supplement: Supplementary file 1 [file OncolRes-34-70837-s001.zip › TSP_OR_70837-s001-figures/Fig S2.tiff]

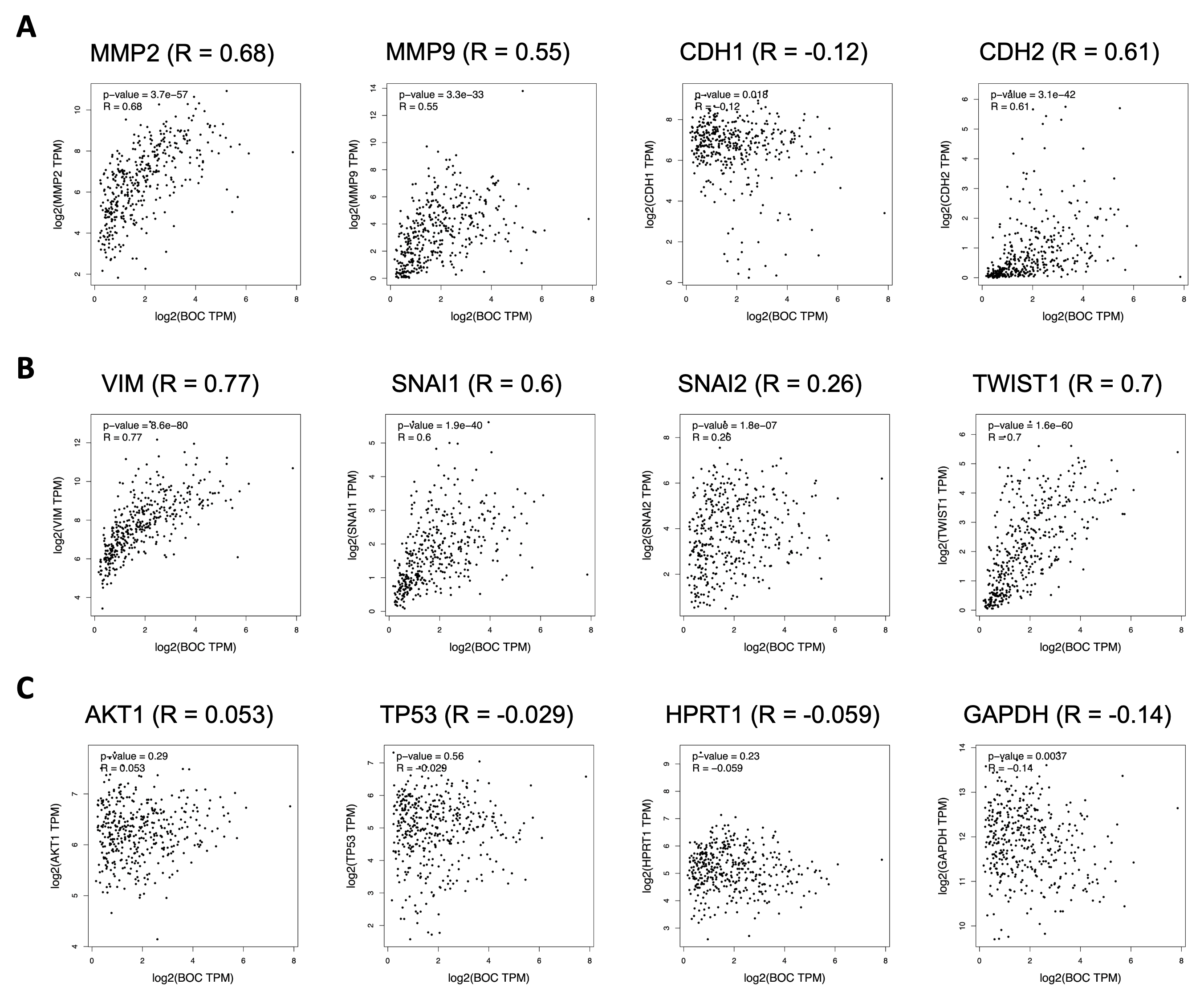

Supplement: Supplementary file 1 [file OncolRes-34-70837-s001.zip › TSP_OR_70837-s001-figures/Fig S3.tiff]

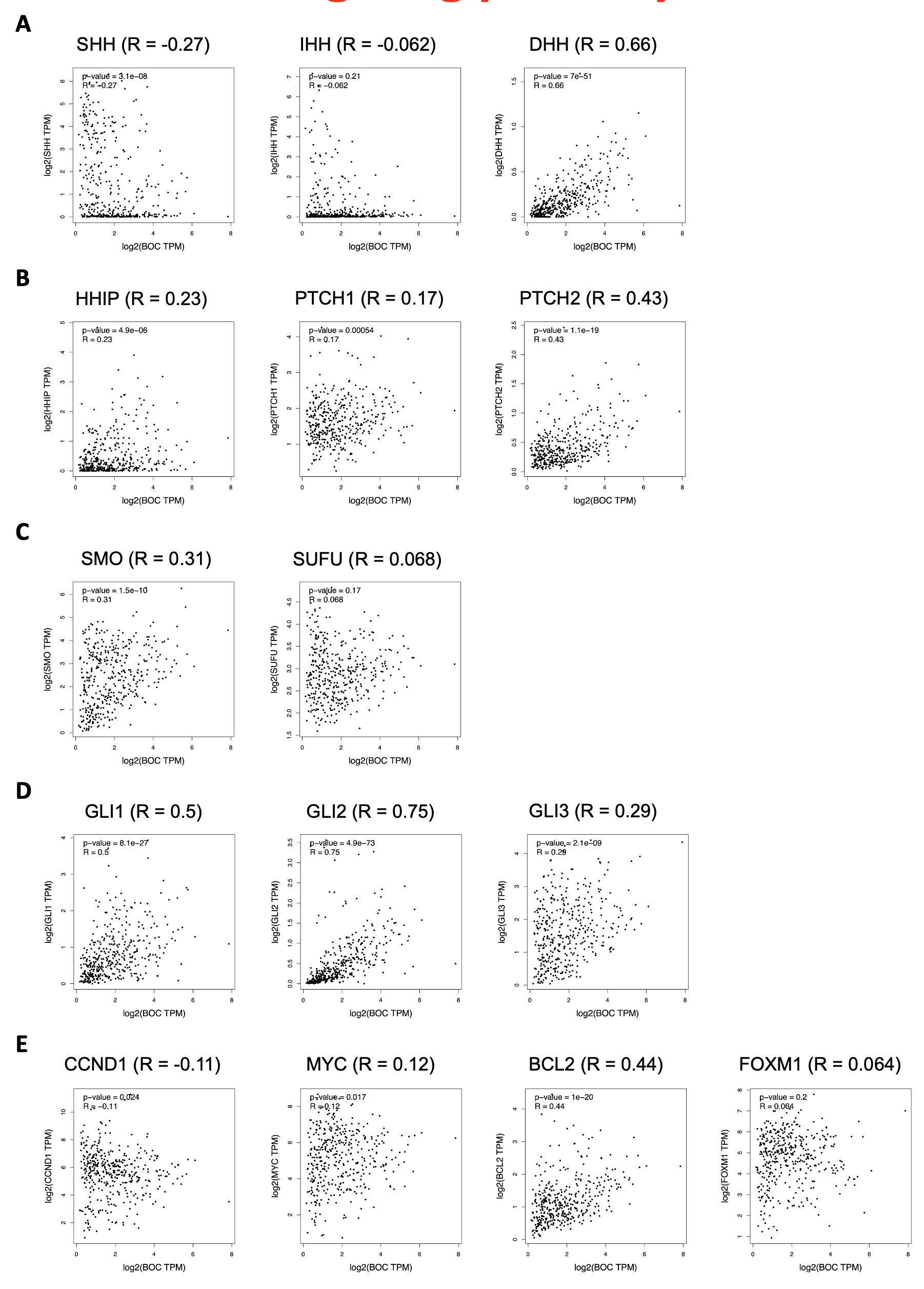

Supplement: Supplementary file 1 [file OncolRes-34-70837-s001.zip › TSP_OR_70837-s001-figures/Fig S4.tiff]

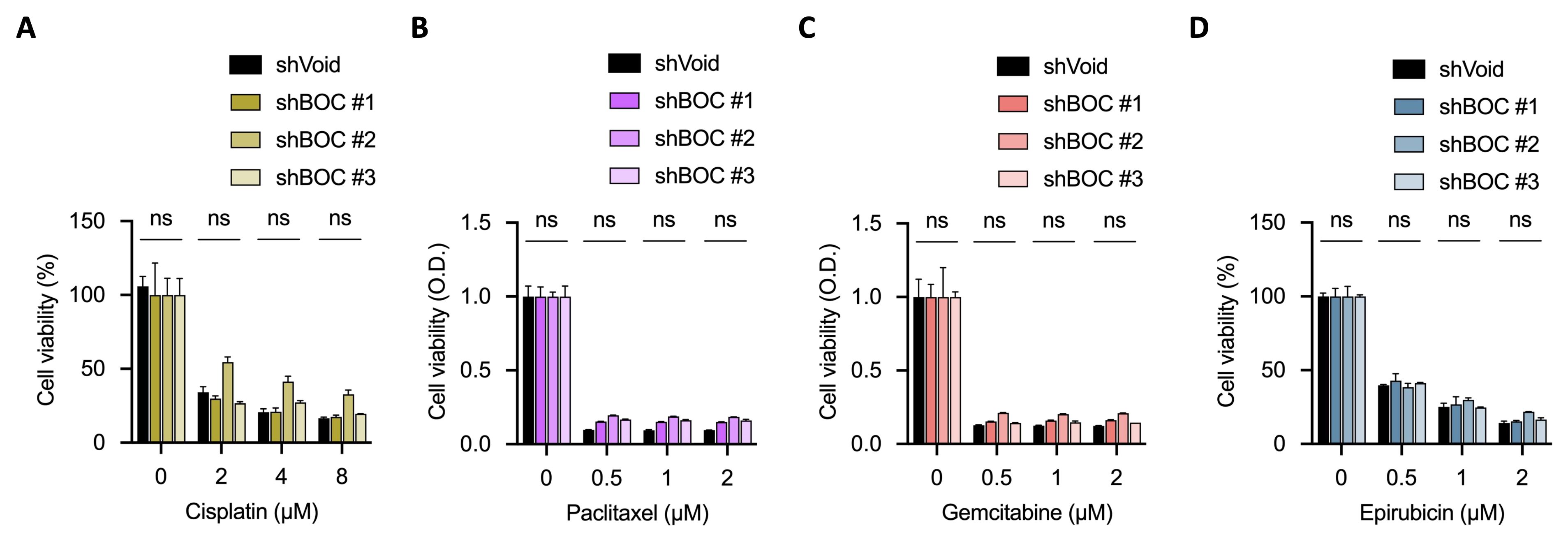

Supplement: Supplementary file 1 [file OncolRes-34-70837-s001.zip › TSP_OR_70837-s001-figures/Fig S5.tiff]

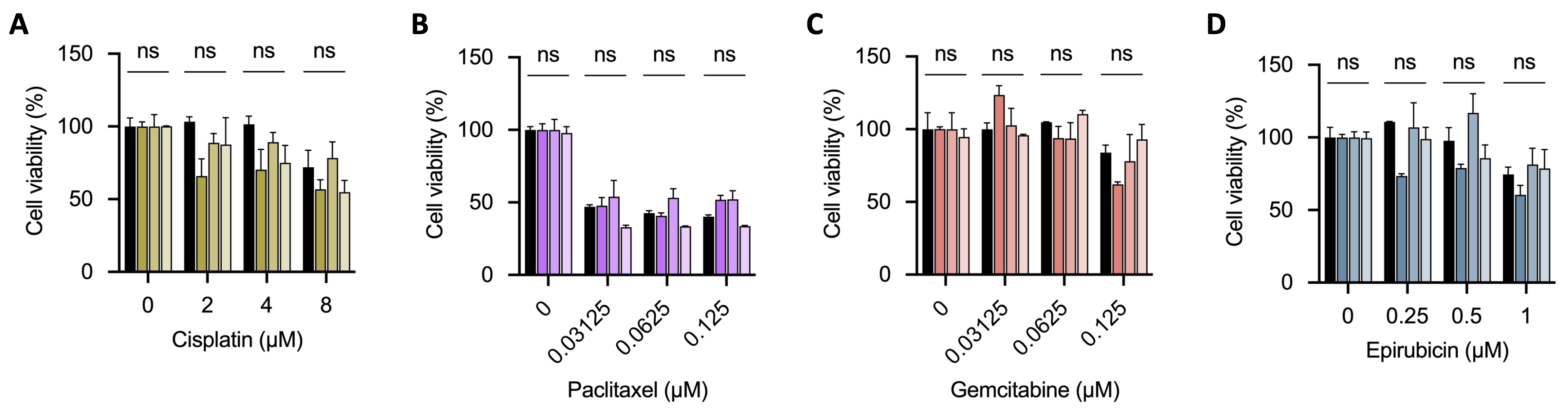

Supplement: Supplementary file 1 [file OncolRes-34-70837-s001.zip › TSP_OR_70837-s001-figures/Fig S6.tiff]

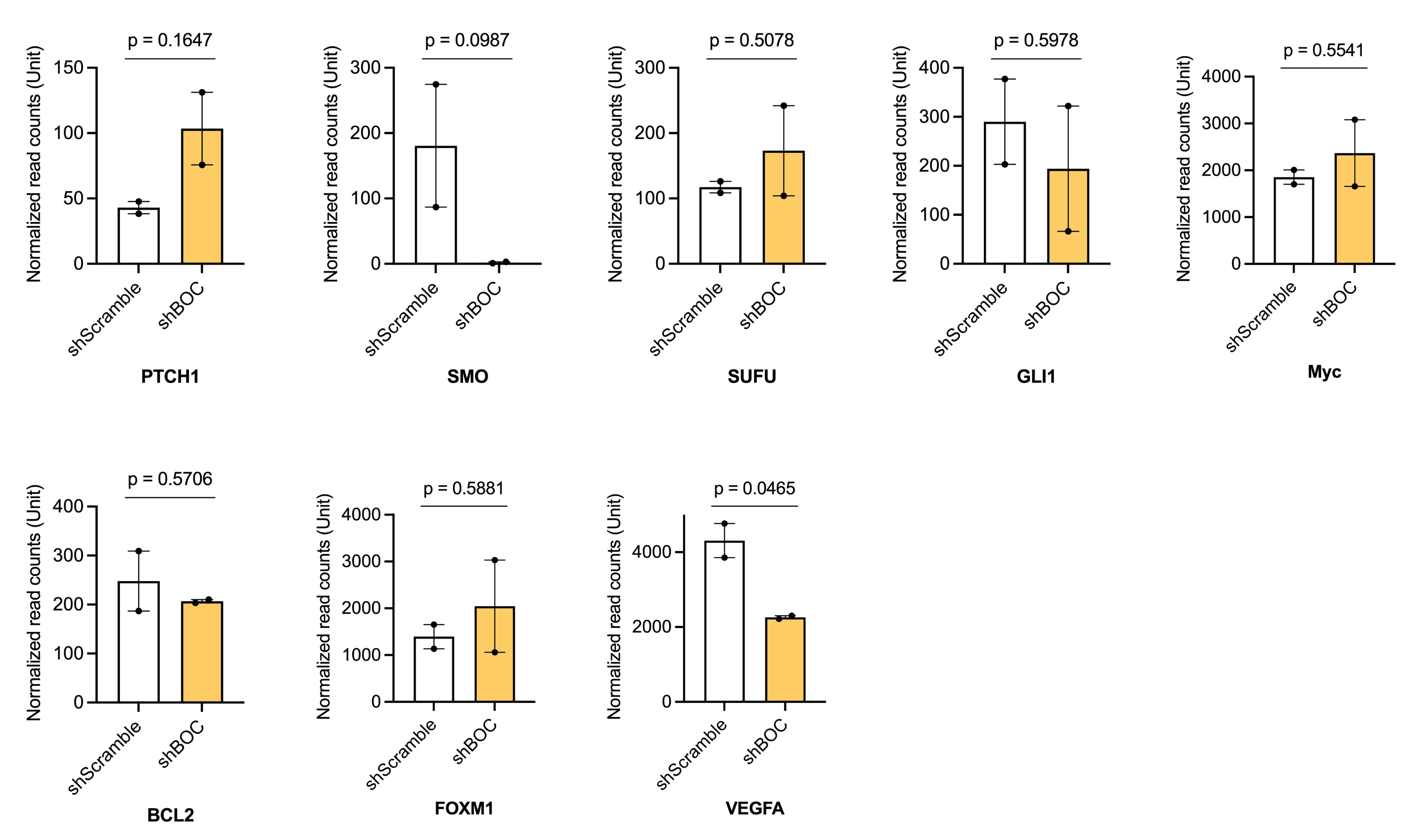

Supplement: Supplementary file 1 [file OncolRes-34-70837-s001.zip › TSP_OR_70837-s001-figures/Fig S7.tiff]

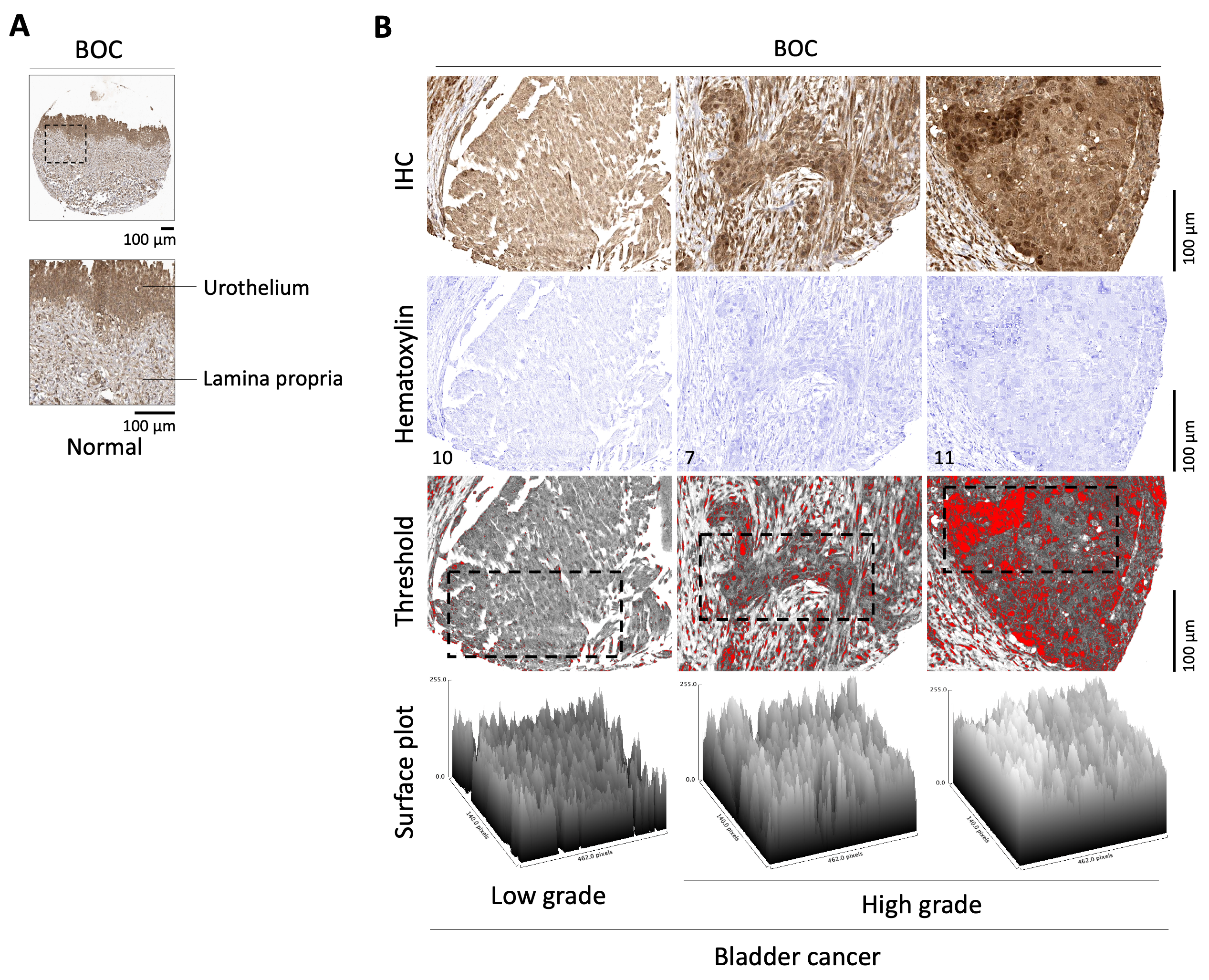

Supplement: Supplementary file 1 [file OncolRes-34-70837-s001.zip › TSP_OR_70837-s001-figures/Fig S8.tiff]

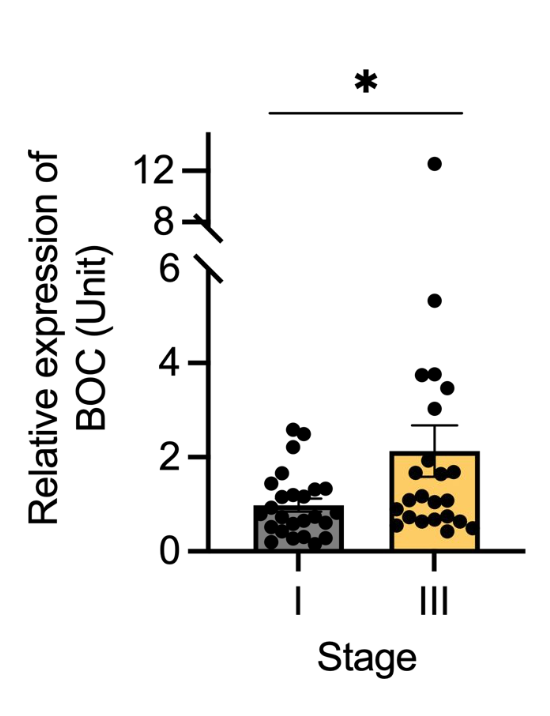

Supplement: Supplementary file 1 [file OncolRes-34-70837-s001.zip › TSP_OR_70837-s001-figures/Fig S9.tiff]
